# Supplementary material for: Computer-aided discovery of connected metal-organic frameworks
Source: Nat Commun. 2019 Aug 9;10:3620. doi: 10.1038/s41467-019-11629-4 (PMC6689093; doi:10.1038/s41467-019-11629-4)
Supplement: Supplementary file 1 — Supplementary Information [file 41467_2019_11629_MOESM1_ESM.pdf]

Supplementary Information

Computer-aided Discovery of Connected Metal-Organic Frameworks

Ohmin Kwon[1,3] and Jin Yeong Kim[2,3] et al.

[1]Department of Chemical and Biomolecular Engineering, Korea Advanced Institute of Science and Technology (KAIST), Daejeon, Republic of Korea

[2]Department of Chemistry, School of Natural Science, Ulsan National Institute of Science and Technology (UNIST), Ulsan 44919, Republic of Korea

[3]These authors contribute equally: Ohmin Kwon, Jin Yeong Kim.

* e-mail: hoirimoon@unist.ac.kr; jihankim@kaist.ac.kr

Supplementary Note 1. Details of algorithm to screen MOF@MOF pairs

In developing our MOF@MOF generation algorithm, two main assumptions were made.

1) The 2D lattice parameters between the surfaces of two different MOF should be matched to be energetically stable (lattice matching condition).

2) The metal nodes of one MOF and the organic ligands of the second MOF should be in close physical proximity to one another to form the ideal interface of MOF@MOFs (chemical connection points matching condition).

Our algorithm consists of three parts: 1) preparation for 2D cleaved MOF structures from the CSD MOF Subset database[1] 2) 2D lattice matching test 3) chemical connection points matching test

Preparation for 2D cleaved MOF structures from CSD MOF Subset database.

The CSD MOF Subset database which consist of 89,484 MOF structures was obtained using program called ConQuest[2] and the solvents were removed by the script supplied by previous paper[1]. For 2D lattice parameter matching, 3D MOF structures have to be cleaved into the surfaces which have 2D lattice parameters. Cleave Surface function in Materials Studio[3] was used to make the 2D surface structures and perl script using MaterialsScript API was used to automate the generation of thousands of 2D surface structures (Supplementary Figure 1 left). Although surface cleave function can generate infinite types of planes in principle, 3D structures were cleaved to generate only the common planes depending on crystal system (as complex planes would be more difficult to synthesize experimentally as well). For simple 2D lattice matching, only cubic, hexagonal and tetragonal cells were used among the whole 3D MOFs because the 2D lattice shapes of these crystal systems are relatively simple

(square or rhombus, see Supplementary Table 1). Cubic MOFs were cleaved with (001) and (111) plane. Hexagonal and Tetragonal cell were cleaved with (001) plane. After cleaving the surfaces, normal vectors of cleaved surfaces were aligned with Cartesian z axis and vacuum space of 15 Å was added above the surface to generate slab 2D cleaved MOF structures for 2D lattice matching (Supplementary Figure 1 right).

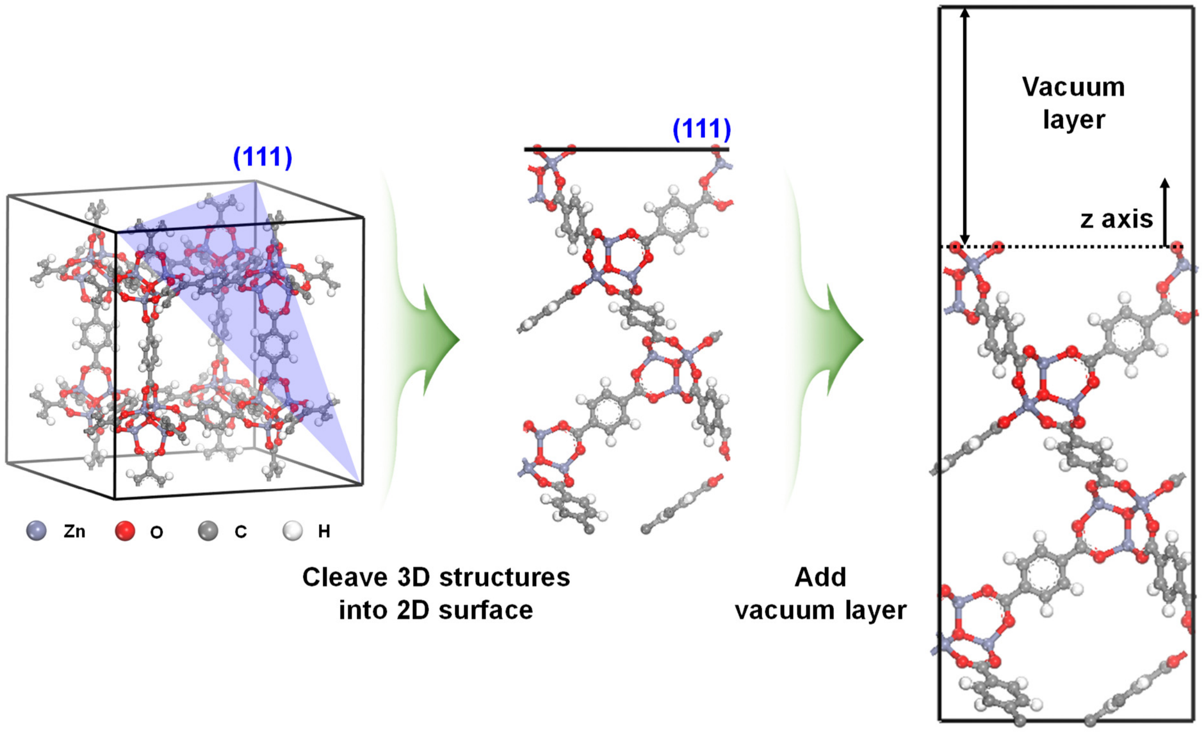

Supplementary Figure 1 | Process to simplify 3D structures to 2D cleaved structures. (an example of MOF-5 structure cleaved on (111) surface)

2D lattice parameter matching test

To determine two MOFs with similar lattice parameters, an algorithm similar to our previous work on hetero-interpenetration was used[4]. Our algorithm checks to see if the ratio of the 2D lattice parameters can be of integer numbers (e.g. 1.0, 2.0, 3.0) with a 3% of error

threshold. The cutoff threshold is somewhat chosen arbitrarily and we surmise that certain pairs of MOFs with larger error can still connect due to flexibility[5]. Supplementary Table 1 summarizes the types of symmetry that were used to compare one MOF against the other MOF.

Symmetry (plane)<br>Shape of 2D cell	Cubic (001)	Cubic (111)	Hexagonal (001)	Tetragonal (001)
Cubic (001)	○	X	X	○
Cubic (111)		○	○	X
Hexagonal (001)			○	X
Tetragonal (001)				○

Supplementary Table 1. Matching pairs in 2D lattice parameter matching test

Chemical connection points matching test

Generation of chemical connection points

To form stable interface between two different MOFs, the metal nodes of one MOF should be connected with the organic ligands of the other MOF at the interface. Thus, the information

of atoms from metal nodes and from organic linkers is necessary for the chemical connection points matching procedure. To this end, 2D slab structures were simplified into chemical connection points to contain only the atoms involved in the coordination bonds at the interface. For all the candidate MOFs, the oxygen atoms of the carboxylic acid were selected as one of the atoms for potential chemical connection points and all of metal atom types were selected as possible candidates for chemical connection points from the metal clusters. First, the information of metal atoms from 2D slab structure generated by previous step were stored to screen the structures which have carboxylic acid and generate the chemical connection points. The information includes the coordinates and bond information of metal atoms. Next, the information of the atoms directly connected with metal atoms called "first connected atoms" were extracted. In the same way, the information of the atoms directly connected with first connected atoms called "second connected atoms" were stored. Except for these atoms (i.e. metal atoms, first connected atoms and second connected atoms), the remaining atoms were deleted to simplify 2D slab structures (Supplementary Figure 2a). From this information, the structures which contain carboxylic acid linker were screened by investigating the existence of COO moiety from carboxylic acid and the oxygen pairs of a carboxylic acid and the metal atom pairs were classified by investigating bond connection information (Supplementary Figure 2b).

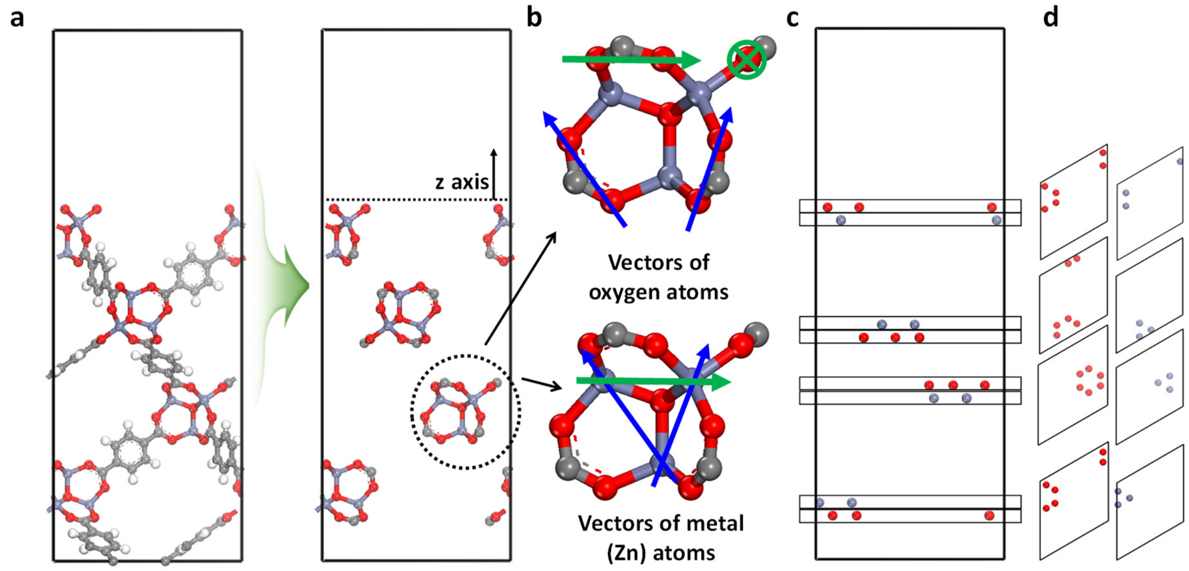

Supplementary Figure 2 | Process to generate “chemical connection points” from 2D cleaved structures (example of MOF-5 structure cleaved on the (111) surface). **a,** Removal of unnecessary atoms from the 2D cleaved structure of MOF-5. **b,** Vectorization of the atom pairs (the green arrows are parallel to cleaved plane whereas the blue arrows are not). **c,** Atoms from the green vectors which are on the cleaved plane **d,** Chemical connection points are generated from each layers of atoms from **c**. (top view)

Next, the vectors from one atom of an oxygen (or metal) atom pair to the other atom were calculated and these vectors were used to check whether the atoms of a pair are on the cleaved plane. If any vectors are not perpendicular to normal vector of cleaved plane parallel to z axis (with 5 degrees of error threshold), the atom pairs were excluded from the chemical connection points (Supplementary Figure 2b-2d). For example, the vectors represented as green arrows in Supplementary Figure 2b were accepted for atom pairs of chemical connection points and the vectors represented as the blue arrows were excluded from chemical connection points. If some two pairs of atoms are within 1 Å along the z axis, two pairs are regarded with being on the

same slice (Supplementary Figure 2c). Finally, several slices of oxygen (or metal) chemical connection points were generated from the 2D slab structures (Supplementary Figure 2d).

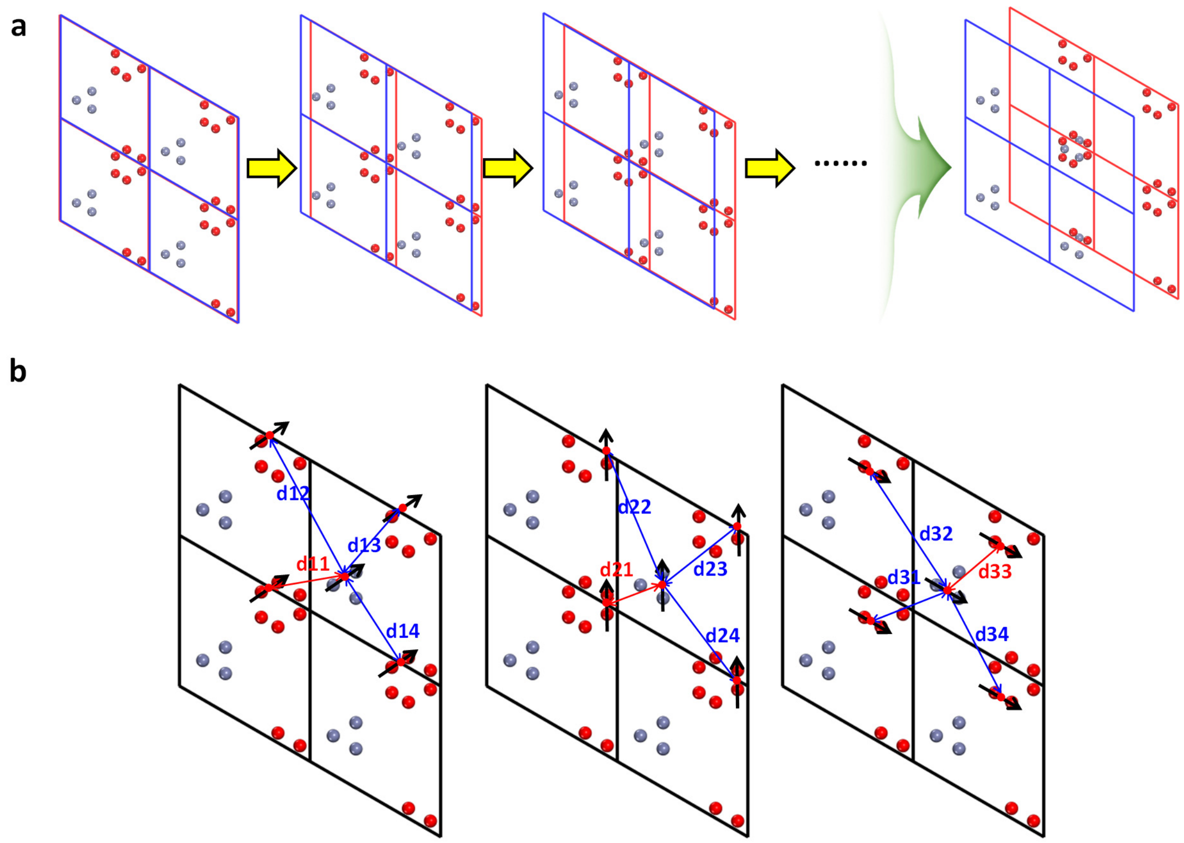

Supplementary Figure 3 | Process to optimize the positions of chemical connection points.

Chemical connection points matching was conducted with these simplified structures with just the relevant atoms. First, 2D supercell was generated using the results from the lattice parameter matching. Next, using the vectors from the previous section, the midpoint of the vector (i.e. center of mass of two atoms) was calculated and used to determine the distances between the atom pairs between the first and the second MOFs. Subsequently, two 2D supercells that contain the chemical connection points were overlapped by putting the origins on the same position (Supplementary Figure 3a left most). And then, The distance (d_{ij})

between midpoints of the vector which belong to different MOF (and have parallel vectors) were calculated for every i and j. (i: index of atom pairs from first MOF, j: index of atom pairs from the second MOF parallel to the i atom pair) (Supplementary Figure 3b red and blue arrow). For every index i, minimum distance ($\min(d_{ij})$) (Supplementary Figure 3b red arrow) was found and $\mathrm{D} = \sum_i (\min(d_{ij}))^2$ (the summation of square distance for red arrow) was calculated. Once a D value was calculated, the second 2D supercell which contains chemical connection points was moved 1 Å along a lattice vector direction. Afterwards, a new D value was calculated in the exactly same way. This procedure was repeated until the second supercell traversed through all of grid points with an interval size of 1 Å (Supplementary Figure 3a middle). Finally, the position where D value is minimum was obtained (Supplementary Figure 3a right most). After the first search, similar search with a more refined grid size (0.01 Å) was conducted to enhance our accuracy.

At the optimal position, the number of connected pair per unit area was calculated, where this number was incremented when two atom pairs were parallel and within 5 Å of one another (this number can vary considering linker strain).

From the results, the candidate pairs with the number of connected pairs smaller than 50% of the total available chemical connection points were excluded from the final candidate set due to potential chemical instability. Final candidate pairs were tabulated in an attached Excel file with the number of connected pair per unit area and the corresponding $\mathrm{D} = \sum_i (\min(d_{ij}))^2$ values.

Among the candidate MOFs that passed the chemical connection points matching procedure, there were disordered MOFs, and these MOFs were omitted for experimental synthesis.

Supplementary Note 2. Screening results of MOF-5

MOF-5 was cleaved into (001) plane and (111) plane and in the case of (001) plane, two kinds of 2D unit cells were used in the lattice parameter matching. The first one is a square 2D unit cell with 25.832 Å lattice (Supplementary Figure 4a) and the second one is a square primitive 2D unit cell with 18.266 Å of lattice constants (Supplementary Figure 4b). 2D rhombus unit cell of MOF-5 (111) plane with 18.266 Å and 120˚ degrees of lattice parameters was used in lattice parameter matching with candidate MOFs.

After lattice matching, chemical connection points matching tests were conducted with the candidate MOFs. It was found that 188 of cubic (001) surface and two tetragonal (001) surface can be connected with (001) surface of MOF-5 and 188 of cubic (111) surface and 18 of hexagonal (001) surface can be connected with (111) surface of MOF-5.

Among the final candidate structures, many were duplicate MOF-5 structures and HKUST-1 with different refcodes. The full refcodes of final candidate MOFs are tabulated in Supplementary Data 1 (Excel file).

Beyond just the MOF-5 case, a general screening was conducted for different MOF pairs. The results of chemical connection points matching in the general case was tabulated in Supplementary Data 1 (Excel file).

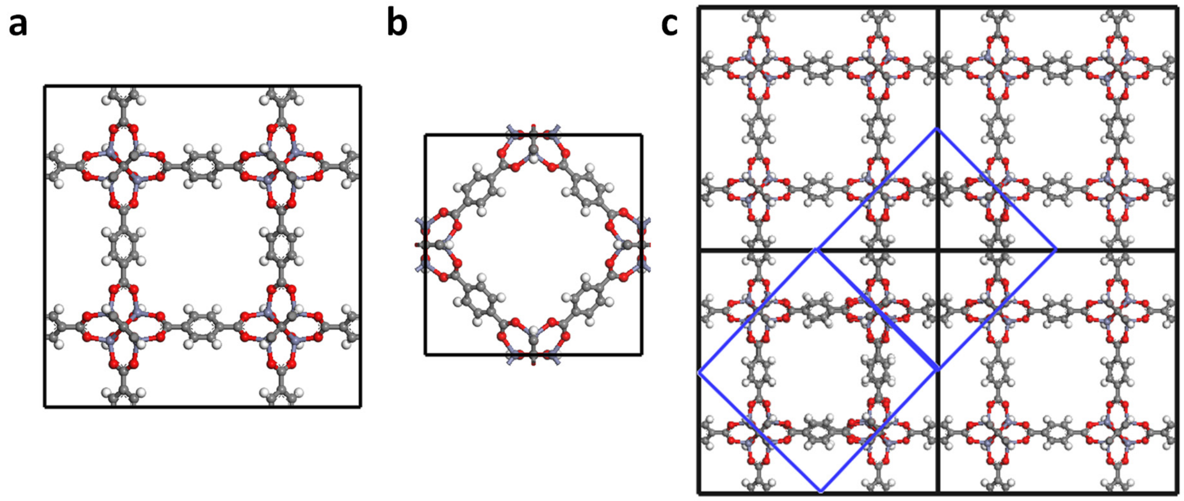

Supplementary Figure 4 | Top view of 2D lattice of MOF-5 (001) plane. **a,** conventional 2D lattice of MOF-5 **b,** primitive 2D lattice of MOF-5 **c,** relationship betweentwo different 2D lattice

Supplementary Note 3. Discussion on model structures of HKUST-1/MOF-5

Chemical connection points matching of HKUST-1/MOF-5 along the (111) plane.

MOF-5 and HKUST-1 were cut into (111) surface and the chemical connection points were generated by our algorithm (Supplementary Figure 5). There are two different combination: 1) MOF-5 (Zn) and HKUST-1(O) 2) MOF-5(O) and HKUST-1(Cu). Both combination turned out to be well matched within our distance threshold of 5 Å. The distance between the metal and the oxygen pairs in 2) is shorter than that of 1) and as such might be more stable.

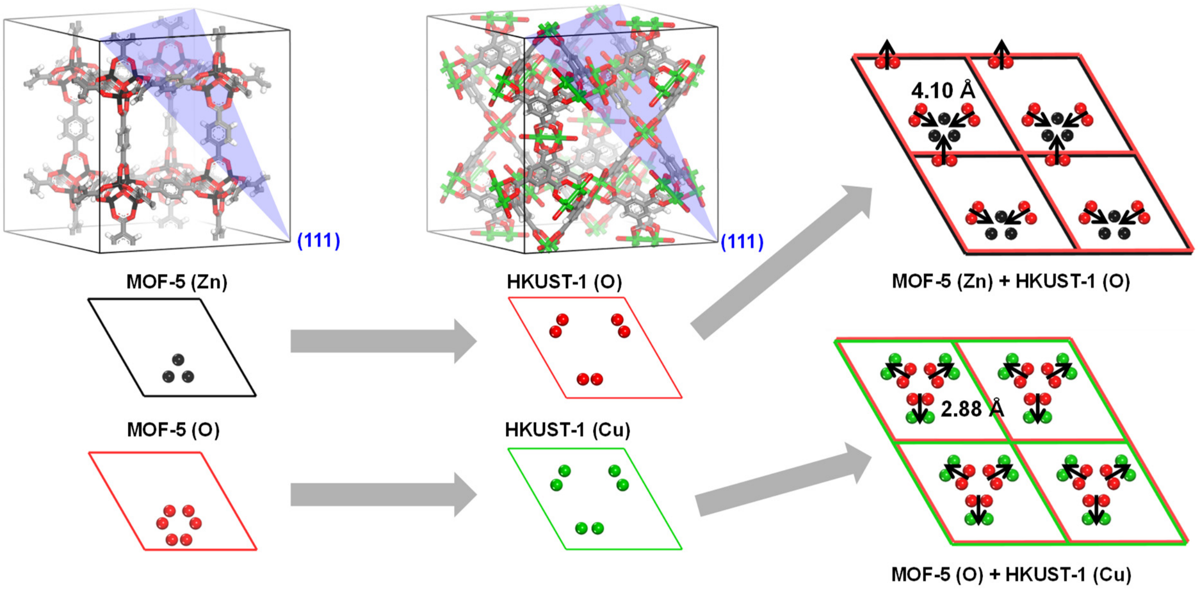

Supplementary Figure 5 | Results of chemical connection points matching on HKUST-1/MOF-5 system along the (111) plane.

Discussion on computational structures of HKUST-1/MOF-5 system

Four different structural models of HKUST-1/MOF-5 system can be proposed from final configuration of chemical connection point matching : model (1) MOF-5(Zn)/HKUST-1(O) along (001) plane; model (2) MOF-5(O)/HKUST-1(Cu) along (001) plane; model (3) MOF-5(Zn)/HKUST-1(O) along (111) plane; model (4) MOF-5(O)/HKUST-1(Cu) along (111) plane.

In the case for model (1), a pair of Zn atoms can form coordination bonds with two pairs of carboxylic acid (btc) when we just consider chemical connection points matching (Supplementary Figure 6a, 6b). However, considering the 3D structures of two MOFs and bonding environment of Zn atoms of MOF-5, which form four bonds, only a pair of oxygen among the two oxygen pair near the Zn atom pair should be used to form the interface bonding due to stability. Thus a btc linker should be removed from a pair of zinc atoms and modulator molecules like acetic acid or solvent molecules should be attached to the vacancy site (Supplementary Figure 6c). But in this case, the available space from the vacancy defect is too

small to fit in the acetic acid molecules or a large solvent (Supplementary Figure 6c right) and will lead instability. Moreover, there should be a large strain from the btc linker making it difficult to form new bonding at the interface (Supplementary Figure 6c left).

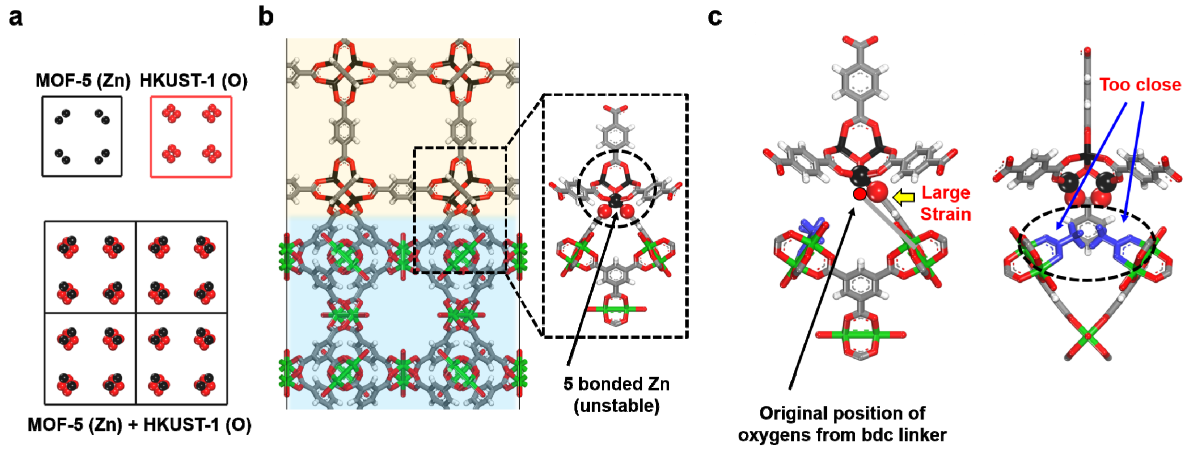

Supplementary Figure 6 | Computational structures of (001) HKUST-1/MOF-5 system from chemical connection points (Model (1) : MOF-5 (Zn) + HKUST-1(O)). **a,** Matched chemical connection points. **b,** 3D structures from chemical connection points. The yellow and green region indicate the space for MOF-5 and HKUST-1, respectively. **c,** Same as **b**. except four bonds from a zinc atom.

On the other hand in model (2), the copper atoms of HKUST-1 are connected with oxygen atoms of bdc linker from MOF-5 and have almost perfect matching of connection points (Supplementary Figure 7a). Considering only the 2D chemical connection points, vertical connection of bdc linker on the Cu dimer can be thought as a possible structure (Supplementary Figure 7b). However, given that the Cu paddlewheel structure is connected with only three carboxylic acids in this case, the more realistic model can be constructed as follows (Supplementary Figure 7c). The model structure in Supplementary Figure 7c have interface of Cu paddlewheel cluster connected with two btc linkers from the original HKUST-1 part, a

solvent or modulator carboxylic acid and a bdc linker which can connect between HKUST-1 and MOF-5. In model (2), the linker strain will be smaller than model (1) because bdc linker can move more freely than the btc linker. But the important point that we want to emphasize here is that both the model (1) and model (2) will yield the same MOF@MOF structures as the atomic positions away from the boundary will be exactly the same in both cases (Supplementary Figure 6b, 7b and 7c).

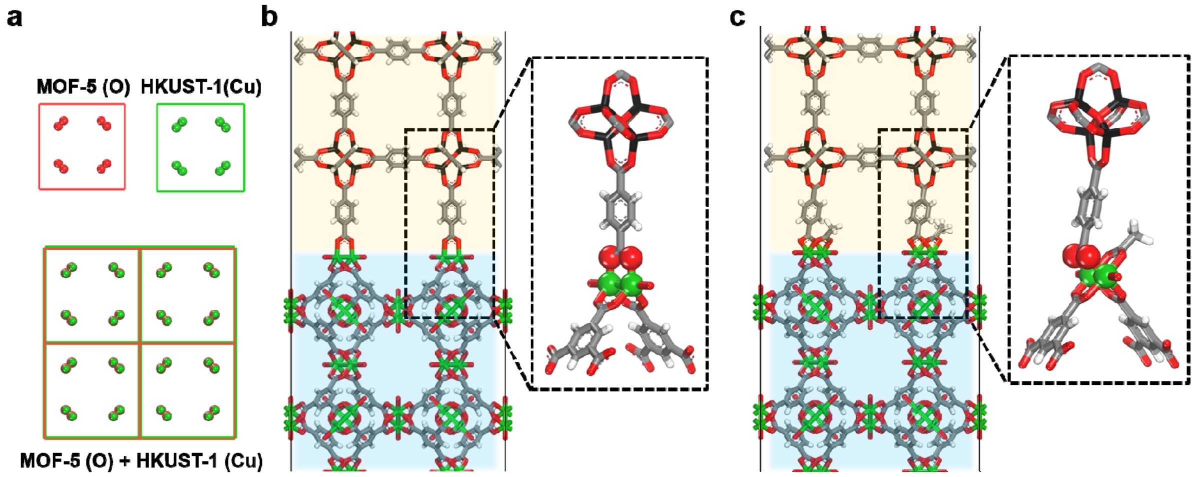

Supplementary Figure 7 | Computational structures of (001) HKUST-1/MOF-5 system from chemical connection points (Model (2) : MOF-5 (O) + HKUST-1(Cu)). **a,** Matched chemical connection points **b,** 3D structures from chemical connection points **c,** Same as **b** except Cu paddlewheel bonded with four carboxylate. In **b** and **c,** the yellow and green region indicate the space for MOF-5 and HKUST-1, respectively.

In a similar way, (111) HKUST-1/MOF-5 model of (3) and (4) have the same conclusion as the previous section. In model (3), zinc atoms of MOF-5 are connected with oxygen atoms of btc linkers from HKUST-1 (Supplementary Figure 8a). And in model (4), copper atoms of HKUST-1 are connected with oxygen atoms of bdc linkers from MOF-5 (Supplementary

Figure 9a). From the chemical connection points matching, zinc atom pairs of MOF-5 and oxygen atom pairs of HKUST-1 are separated by around 4.10 Å along the (111) plane in model (3) (Supplementary Figure 8a). On the other hand, copper atom pairs of HKUST-1 and oxygen atom pairs of MOF-5 are separated by 2.88 Å, which is shorter distance along the (111) plane in model (4) (Supplementary Figure 9a). Moreover, in model (3), it is difficult to connect oxygen atoms with zinc atoms by bending the btc linkers because the btc linkers have already been connected with another metal cluster (Supplementary Figure 8b). From these two reasons (i.e. distance and linker flexibility), model (4) can be thought as more reasonable model for (111) HKUST-1/MOF-5 case and was adopted as our computational model structure. Similar to the (001) models, the atomic positions away from the interface remain exactly the same for both models of (111) HKUST-1/MOF-5 (Supplementary Figure 8b and 9b).

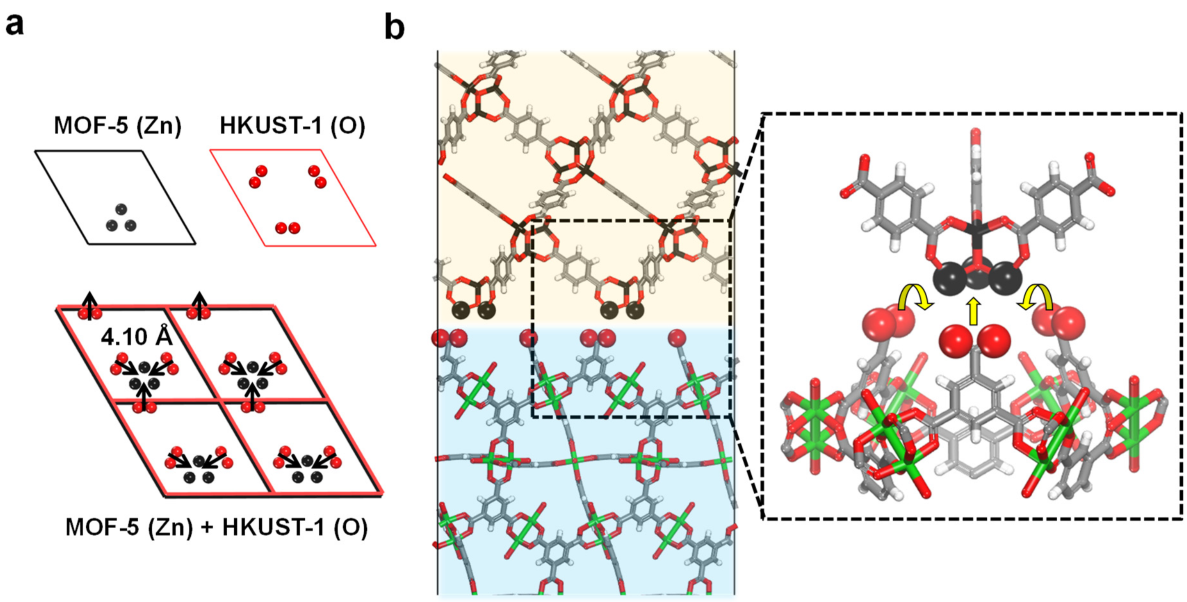

Supplementary Figure 8 | Computational structures of (111) HKUST-1/MOF-5 system from chemical connection points (Model (3) : MOF-5 (Zn) + HKUST-1(O)). **a,** Matched chemical connection points **b,** 3D structures from chemical connection points. The yellow and green region indicate the space for MOF-5 and HKUST-1, respectively.

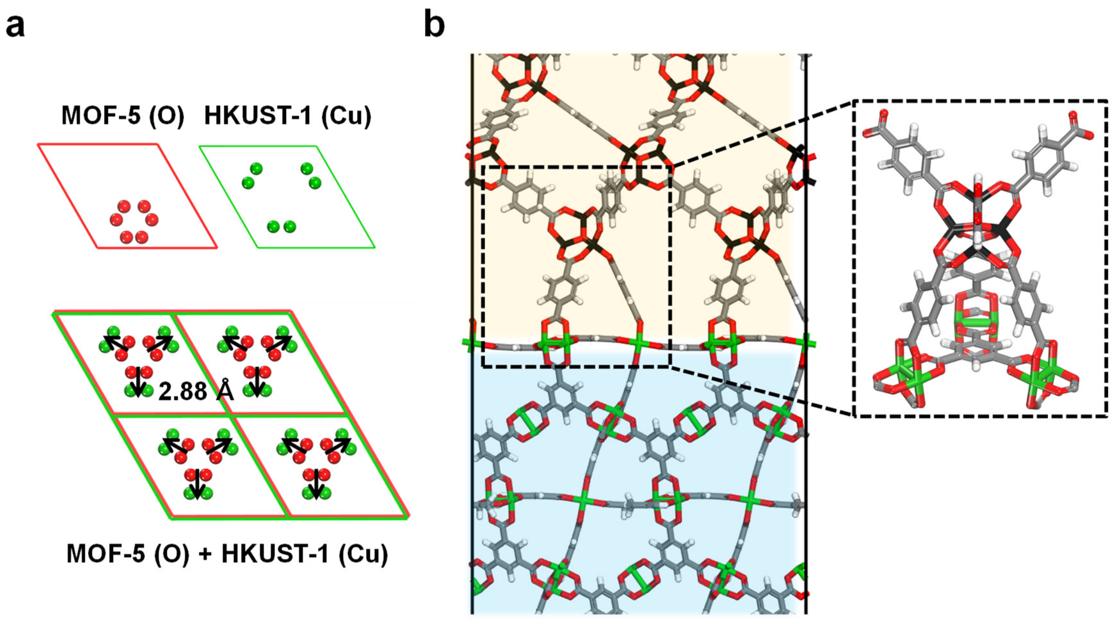

Supplementary Figure 9 | Computational structures of (111) HKUST-1/MOF-5 system from chemical connection points (Model (4) : MOF-5 (O) + HKUST-1(Cu)). **a,** Matched chemical connection points **b,** 3D structures from chemical connection points. The yellow and green region indicate the space for MOF-5 and HKUST-1, respectively.

Supplementary Note 4. Energy calculation using Density Functional Theory (DFT) to estimate stability of interface between HKUST-1 and MOF-5

Energy stabilization from new interface bonding was estimated by calculating the binding energy of a bdc linker on the Cu paddlewheel part and energy penalty from linker strain by calculating energy difference between strained cluster and original MOF cluster (Supplementary Figure 10-11). The binding energy between bdc linker of MOF-5 and HKUST-1 (001) and (111) surface was calculated with the Supplementary Equation 1 and 2 respectively

$$E_{binding(001)} = E_{(H_2O)_2(Cu)_2(btc)_2(bdc)(\text{acetic})} - (E_{(H_2O)_2(Cu)_2(btc)_2(acetic)} + E_{bdc}) \quad (1)$$

where $E_{(H_2O)_2(Cu)_2(btc)_2(bdc)(\text{acetic})}$ is total energy of a paddlewheel metal cluster connected

with 2 btc linkers, a bdc linker and a acetic acid (Supplementary Figure 10a), $E_{(H_2O)_2(Cu)_2(btc)_2(acetic)}$ is total energy of a paddlewheel metal cluster with only 2 btc linkers, a acetic acid and a vacancy site (Supplementary Figure 10b) and E_{bdc} is total energy of a bdc linker (Supplementary Figure 10c).

$$E_{binding(111)} = E_{(H_2O)_2(Cu)_2(btc)_3(bdc)} - (E_{(H_2O)_2(Cu)_2(btc)_3} + E_{bdc}) \qquad (2)$$

where $E_{(H_2O)_2(Cu)_2(btc)_3(bdc)}$ is total energy of a paddlewheel metal cluster connected with 3 btc linkers and a bdc linker (Supplementary Figure 11a), $E_{(H_2O)_2(Cu)_2(btc)_3}$ is total energy of a paddlewheel metal cluster with only 3 btc linkers and a vacancy site (Supplementary Figure 11b) and E_{bdc} is total energy of a bdc linker (Supplementary Figure 11c).

The strain energy of HKUST-1 part and MOF-5 part were obtained by calculating the energy difference between the original structure (Supplementary Figure 10d, 10f, 11d and 11f) and the structure where a linker was moved to make a connection at the interface (Supplementary Figure 10e, 10g, 11e and 11g). The strain energy was calculated with the Supplementary Equation 3

$$E_{strain} = E_{\text{strained structure(scf)}} - E_{\text{original structure(relaxed)}} \qquad (3)$$

All the calculated values and the net energy per unit area in each case considering the number of interface bonding and 2D lattice area with conventional unit of interface energy [eVÅ^{-2}] are tabulated in Supplementary Table 2.

	(001) plane case		(111) plane case	
$E_{binding}$	$E_{\mathrm{Fig.S10a}} - (E_{\mathrm{Fig.S10b}} + E_{\mathrm{Fig.S10c}})$		$E_{\mathrm{Fig.S11a}} - (E_{\mathrm{Fig.S11b}} + E_{\mathrm{Fig.S11c}})$	
	−224.6 $kJmol^{-1}$		−220.8 $kJmol^{-1}$	
E_{strain}	HKUST-1 part	MOF-5 part	HKUST-1 part	MOF-5 part
	$E_{\mathrm{Fig.S10e}} - E_{\mathrm{Fig.S10d}}$	$E_{\mathrm{Fig.S10g}} - E_{\mathrm{Fig.S10f}}$	$E_{\mathrm{Fig.S11e}} - E_{\mathrm{Fig.S11d}}$	$E_{\mathrm{Fig.S11g}} - E_{\mathrm{Fig.S11f}}$
	+ 72.2 $kJmol^{-1}$	+ 51.7 $kJmol^{-1}$	+ 23.7 $kJmol^{-1}$	+ 60.1 $kJmol^{-1}$
E_{net}	− 100.7 $kJmol^{-1}$		− 137.0 $kJmol^{-1}$	
Area	693.6 Å		300.3 Å	
$N_{bonding}$	4		3	
E_{net}	− 0.0060 $eVÅ^{-2}$		− 0.0142 $eVÅ^{-2}$	

Supplementary Table 2. Results of DFT energy analysis at the interface. $N_{bonding}$ means the number of interface bonding between Cu paddlewheel and bdc linker in a 2D unit cell.

In both cases, the net energy difference was negative value due to large bonding energy term relative to the linker strain energy term. Thus, the formation of bonding at the interface to connect different MOFs could be possible and the HKUST-1/MOF-5 MOF@MOF system was adopted as our first candidate pairs to synthesize.

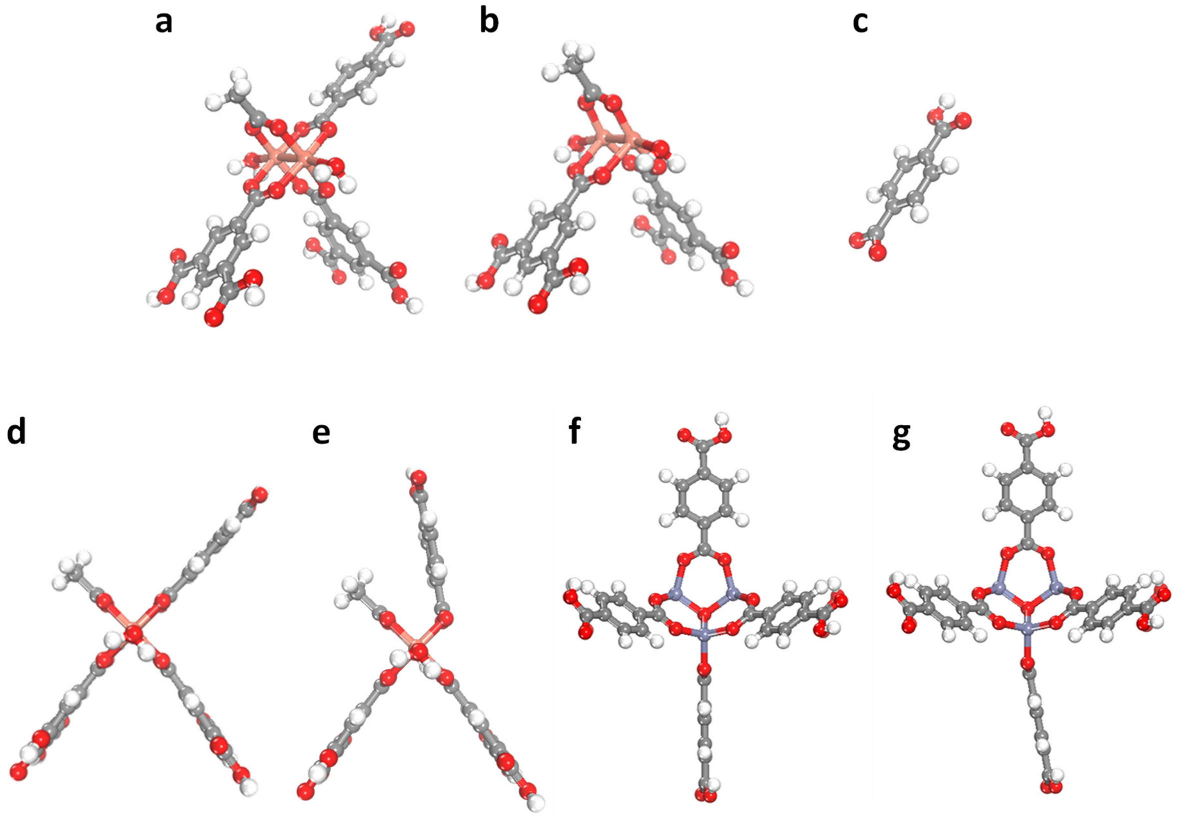

Supplementary Figure 10 | Cluster models of HKUST-1/MOF-5 (001) case to calculate binding energy of bdc (**a-c**), strain energy of HKUST-1 part (**d-e**) and of MOF-5 part (**f-g**). **a,** relaxed 4 bonded Cu paddlewheel cluster (2 btc, 1 bdc and 1 acetic acid) **b,** relaxed 3 bonded Cu paddlewheel cluster (2 btc, 1 acetic acid and a vacancy site) **c,** relaxed 1 bdc linker **d,** relaxed 4 bonded Cu paddlewheel cluster (2 btc, 1 bdc and 1 acetic acid) same as (**a**) but different view. **e,** strained cluster with a bended bdc linker. **f,** relaxed Zn_4O cluster connected with six bdc linkers **g,** strained cluster with a bended bdc linker.

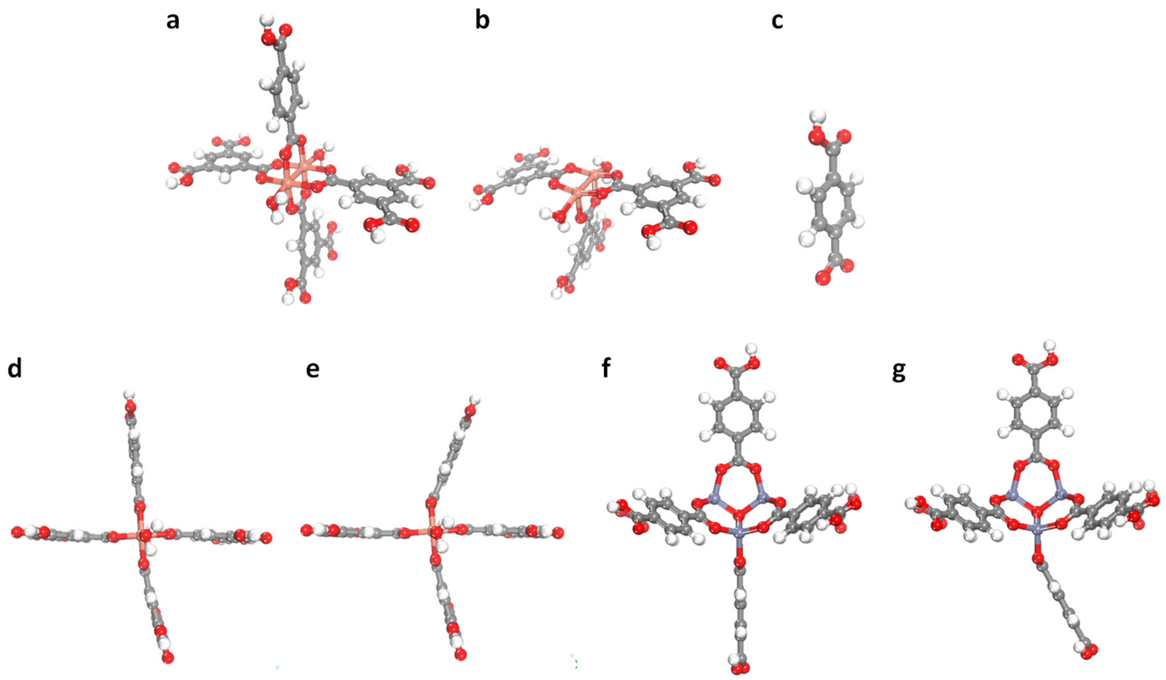

Supplementary Figure 11 | Cluster models of HKUST-1/MOF-5 (111) case to calculate binding energy of bdc (**a-c**), strain energy of HKUST-1 part (**d-e**) and of MOF-5 part (**f-g**). **a,** relaxed 4 bonded Cu paddlewheel cluster (3 btc and 1 bdc) **b,** relaxed 3 bonded Cu paddlewheel cluster (3 btc and a vacancy site) **c,** relaxed 1 bdc linker **d,** relaxed 4 bonded Cu paddlewheel cluster (3 btc and 1 bdc). same as (**a**) but different view **e,** strained cluster with a bended bdc linker. **f,** relaxed Zn_4O cluster connected with six bdc linkers **g,** strained cluster with a bended bdc linker.

Supplementary Methods

Synthesis of Cubic HKUST-1.

Cubic HKUST-1 was prepared by a reported method with minor modifications[6]. $Cu(NO_3)_2 \cdot 2.5H_2O$ (0.472 g, 2.03 mmol) was dissolved in 6 mL of 1:1 H_2O/DMF mixture in a 20 mL vial. Benzene-1,3,5-tricarboxylic acid (H_3BTC) (0.176 g, 0.838 mmol) was completely dissolved in slightly heated ethanol (2.2 mL) with stirring. To Cu nitrate solution, ethanolic ligand solution and glacial acetic acid (12 mL) were added and placed at 55 ºC oven. After 60 hours, the mother liquor quickly decanted and the blue crystals were washed with fresh ethanol. For MOF@MOF synthesis, ethanol washed HKUST-1 crystals were stored in DEF solvent.

Synthesis of MOF-5.

$Zn(NO_3)_2 \cdot 6H_2O$ (0.760 g, 2.55 mmol) and terephthalic acid (0.132 g, 0.795 mmol) were dissolved in 20 mL of DEF in a glass jar. The glass jar was placed at 85 ºC oven. After 4 days, the mother liquor was decanted and the large colorless cubic crystals were washed with fresh DEF and dichloromethane.

Synthesis of Co-bdc[7].

$Co(NO_3)_2 \cdot 6H_2O$ (0.099 g, 0.340 mmol) was dissolved in 2 mL of DMF in a glass jar. Terephthalic acid (0.040 g, 0.240 mmol) was completely dissolved in 3 mL of dimethyl sulfoxide (DMSO). To Cu nitrate solution, DMSO solution was added and heated at 120 ºC for 24 hours. After cooled to room temperature, the mother liquor was decanted and the pink rod crystals were washed with fresh DMSO. For MOF@MOF synthesis, DMSO washed Co-bdc crystals were stored in DEF solvent.

Synthesis of H_6ptei ligand for PCN-68

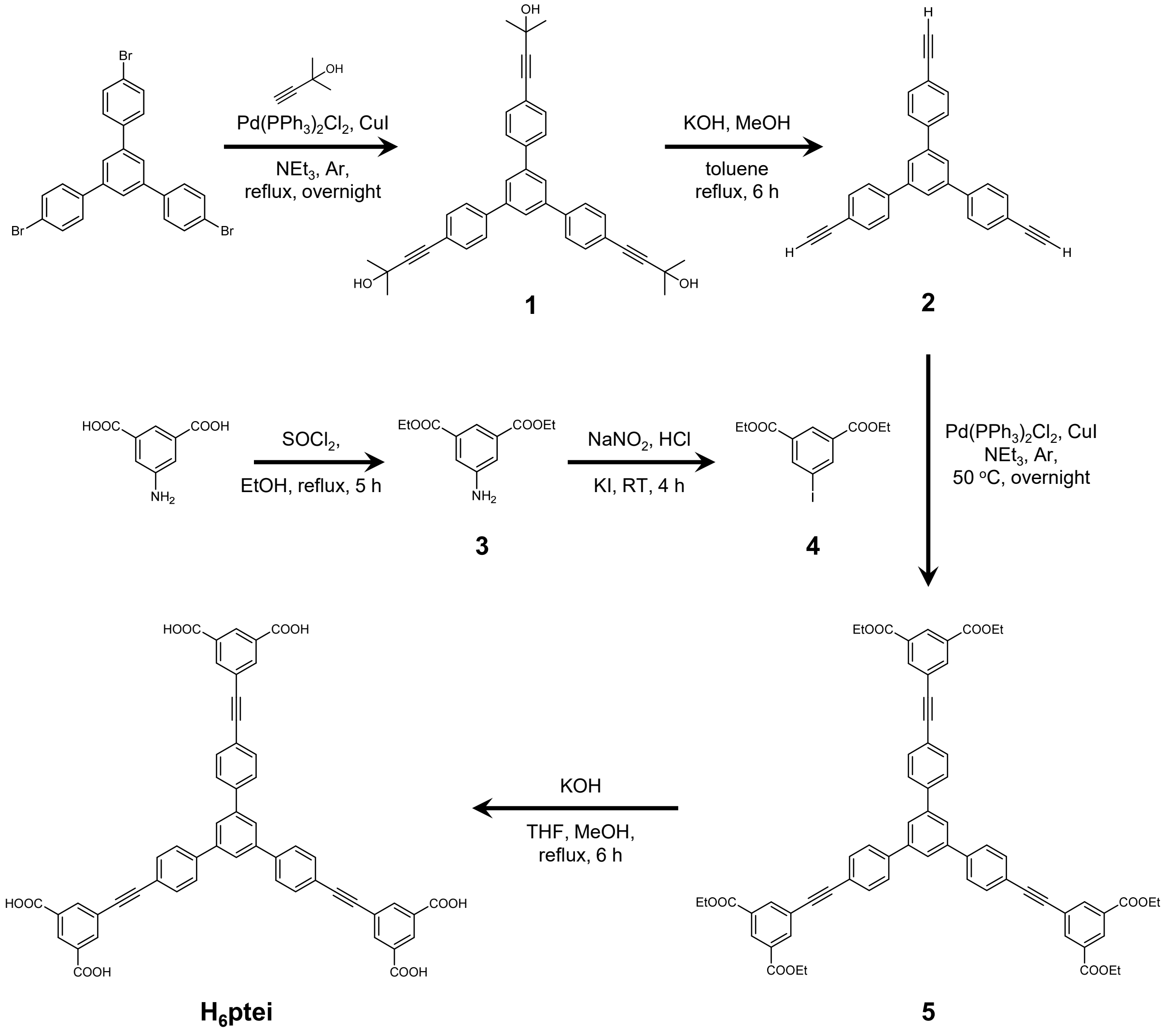

Synthesis of 1,3,5-tri-[4-(3-hydroxy-3-methyl-but-1-ynyl)phenyl]benzene[8](1)

1,3,5-Tri(4-bromophenyl)benzene (5.00 g, 9.21 mmol) was dissolved in distilled triethylamine (TEA) (183 mL) with stirring in a three-necked round-bottom flask. To a stirring solution, copper (I) iodide (0.142 g, 0.746 mmol) and triphenylphosphine (0.192 g, 0.730 mmol) were added. 2-methyl-3-butyn-2-ol (7.3 mL, 86.8 mmol) was added dropwise to the solution. The mixture was bubbled with argon for 30 minutes, then bis(triphenylphosphine)palladium(II) dichloride (0.261 g, 0.372 mmol) was added and the reaction mixture was refluxed overnight under argon atmosphere. After cooling to room temperature, the mixture was filtered and

washed with TEA briefly. The filtrate was diluted with ethyl acetate and washed three times with distilled water. The organic layer was dried over sodium sulfate and filtered. The solvent was removed using a rotary evaporator, and the resulting yellow product was recrystallized from hot toluene. Yield: 4.96 g, 98 %. ^{1}H NMR (400 MHz, $CDCl_3$) δ 7.75 (s, 1H), 7.64 (d, 2H), 7.54 (d, 4H), 1.65 (s, 6H).

Synthesis of 1,3,5-tri(4-ethynylphenyl)benzene[8] (2)

1 (2.87 g, 5.20 mmol) was suspended in toluene (45 mL), to which 17 mL of methanol solution containing potassium hydroxide (2.44 g, 43.7 mmol) was added. Dean-Stark trap was attached with a reflux condenser and the suspension was stirred at 120 °C for 3 hours. After cooling to room temperature, the organic solvent was evaporated using a rotary evaporator. The crude residue was diluted with dichloromethane and washed three times with distilled water. The organic layer was dried over sodium sulfate and filtered. The organic solvent was removed using a rotary evaporator to give a yellow solid. Yield: 1.74 g, 88 %. ^{1}H NMR (400 MHz, $CDCl_3$) δ 7.76 (s, 1H), 7.64 (q, 4H), 3.15 (s, 1H).

Synthesis of diethyl-5-(amino)isophthaloate[9] (3)

5-(amino)isophthalic acid (5.70 g, 31.5 mmol) was dissolved in absolute ethanol (60 mL), to which thionyl chloride (6.80 mL, 93.7 mmol) was added dropwise while stirring at 0 °C. The resulting milky white mixture was refluxed for 5 hours. After cooling to room temperature, the organic solvent was evaporated using a rotary evaporator. The crude residue was dissolved in ethyl acetate and washed with saturated aqueous solution of sodium carbonate. The organic phase was dried over sodium sulfate and filtered. The solvent was removed using a rotary evaporator to obtain a white solid. Yield: 6.71 g, 90 %. ^{1}H NMR (400 MHz, $CDCl_3$) δ 8.06 (t, 1H), 7.52 (d, 2H), 4.37 (q, 4H), 3.90 (bs, 2H), 1.40 (t, 6H).

Synthesis of diethyl-5-(iodo)isophthaloate[9] (4)

A 22 mL of aqueous solution containing sodium nitrite (2.39 g, 34.7 mmol) was added to 32.4 mL of hydrochloric acid (2 M) containing **3** (6.80 g, 28.7 mmol) at 0 ºC and then stirred for 45 minutes. An ice-cold solution of potassium iodide (10.8 g, 65.2 mmol) in distilled water (110 mL) was added dropwise at 0 ºC. Dichloromethane (108 mL) was added and the resulting mixture was stirred at room temperature for 4 hours. The aqueous phase was extracted with dichloromethane three times, and the combined organic layers were washed with sodium thiosulfate solution and brine. The combined organic layers were dried over magnesium sulfate and filtered. After the solvent was removed, the crude yellow product was purified by column chromatography with dichloromethane as eluent to give a pale yellow product. Yield: 6.08 g, 61 %. ^{1}H NMR (400 MHz, $CDCl_3$) δ 8.63 (t, 1H), 8.54 (d, 2H), 4.40 (q, 4H), 1.41 (t, 6H).

Synthesis of hexaethyl tetraethyl 5,5'-((5'-(4-((3,5-bis(ethoxycarbonyl)phenyl)ethynyl) phenyl)-[1,1':3',1''-terphenyl]-4,4''-diyl)bis(ethyne-2,1-diyl))diisophthalate[10,11] (5)

4 (1.61 g, 4.63 mmol), bis(triphenylphosphine)palladium(II) dichloride (0.0661 g, 0.0941 mmol), triphenylphosphine (0.0494 g, 0.188 mmol), and copper (I) iodide (0.0359 g, 0.188 mmol) were placed in 2 neck round bottom flask and degassed in separated flasks by several cycles of vacuum followed by argon backfill. 44 mL of 1:1 degassed tetrahydrofuran/ trimethylamine mixture was added to the flask. **2** (0.500 g, 1.32 mmol) was dissolved in distilled tetrahydrofuran (THF) (10 mL) and bubbled with argon for 20 minutes. A solution of **2** was added dropwise to **4** mixture and the reaction mixture was stirred at 50 ºC under argon atmosphere overnight. After removal of organic solvent, the residue was dissolved in dichloromethane and washed with brine. The organic layer was dried over magnesium sulfate and evaporated using a rotary evaporator. The residual crude yellow product was purified by column chromatography with dichloromethane then ethyl acetate as eluent to give a pale yellow

product. Yield: 1.21 g, 88 %. ^{1}H NMR (400 MHz, $CDCl_3$) δ 8.64 (t, 3H), 8.40 (d, 6H), 7.84 (s, 3H), 7.73 (d, 6H), 7.70 (d, 6H), 4.44 (q, 12H), 1.45 (t, 18H).

Synthesis of 5,5'-((5'-(4-((3,5-dicarboxyphenyl)ethynyl)phenyl)-[1,1':3',1''-terphenyl]-4,4''-diyl)-bis(ethyne-2,1-diyl))diisophthalic acid, H_6ptei[10,11].

5 (1.40 g, 1.34 mmol) was suspended in 126 mL of 1:1 tetrahydrofuran/methanol mixture. To the suspension 63 mL of potassium hydroxide aqueous solution (5 M) was added under stirring and the mixture was refluxed for 4 hours. After cooling to room temperature, the volatile solvents were removed using a rotary evaporator. The residue was dissolved in distilled water and any impurities were removed by centrifugation. The resulting aqueous solution was acidified until a precipitate was appeared by concentrated hydrochloric acid. The precipitate was collected by centrifugation and washed with distilled water several times and recrystallized from DMF/H_2O to yield light yellow products. Yield: 0.798 g, 49.6 %. NMR (400 MHz, DMSO-d_6) δ 8.46 (t, 3H), 8.30 (d, 6H), 8.09 (s, 3H), 8.05 (d, 6H), 7.78 (d, 6H).

Synthesis of PCN-68[10]

H_6ptei (0.0498 g, 0.0572 mmol) and $Cu(NO_3)_2 \cdot 2.5H_2O$ (0.150 g, 0.645 mmol) were dissolved in DMF (7.5 mL) in a 20 mL vial. HBF_4 (240 μL) was added to the solution. The vial was placed at 75 °C oven. After 18 hours, the mother liquor quickly decanted and the resulting green crystals were washed with fresh DMF. For MOF@MOF synthesis, DMF washed PCN-68 crystals were stored in DEF solvent.

Synthesis of UiO-66[12]

Terephthalic acid (0.100 g, 0.602 mmol) was dissolved in 140 mL of DMF containing triethylamine (0.01 mL) in 250 mL round-bottom flask and stirred (600 rpm) for 10 minutes before the addition of acetic acid (20.6 mL). The flask was placed in an oil bath and heated to

120 °C. After the temperature of the oil bath reached 120 °C, 10 mL of DMF solution containing $ZrCl_4$ (0.140 g, 0.601 mmol) was added. After 6 hours, the white powder was collected by centrifugation, washed 3 times with DMF and dried at vacuum oven at room temperature overnight.

Synthesis of UiO-67[13]

$ZrCl_4$ (0.0186 g, 0.0798 mmol) was dissolved in 5 mL of DMF containing acetic acid (1.38 mL). 4,4'-biphenyldicarboxylic acid (0.0193 g, 0.0797 mmol) was dissolved in DMF (5 mL) containing triethylamine (30 μL). Each solution was sonicated for 10 min and combined in 20 mL vial. The combined solution was further sonicated for 10 min and placed at 85 °C oven. After 8 hours, the white powder was collected by centrifugation and washed 3 times with DMF and dried at vacuum oven at room temperature overnight.

Synthesis of MIL-88A[14]

Fumaric acid (0.232 g, 2.00 mmol) and $FeCl_3 \cdot 6H_2O$ (0.541 g, 2.00 mmol) were dissolved in 10 mL of distilled water. The mixture was placed in a Teflon vessel within the autoclave and heated 85 °C for 6 hours. The orange powder was collected by centrifugation and washed 3 times with distilled water.

Synthesis of MIL-88B[15]

Terephthalic acid (0.266 g, 1.60 mmol) and $FeCl_3 \cdot 6H_2O$ (0.432 g, 1.60 mmol) were dissolved in DMF (8 mL) with 2 M NaOH (0.64 mL). The mixture was placed in Teflon vessel within the autoclave, and heated 100 °C for 8 hours with a heating ramp of 3 °C/min. The orange powder was collected by centrifugation and washed 3 times with DMF.

Synthesis of MIL-88C[16]

2,6-naphthalene dicarboxylic acid (2.08 g, 9.62 mmol) and $FeCl_3 \cdot 6H_2O$ (2.56 g, 9.47 mmol) were dissolved in DMF (100 mL). The mixture was placed in a 250 mL round-bottom flask and heated at 130 °C for 18 hours with stirring (300 rpm). The orange powder was collected by centrifugation and washed 3 times with DMF.

Preparation of HKUST-1@IRMOF-18[17]

$Zn(NO_3)_2 \cdot 6H_2O$ (0.200 g, 0.672 mmol) and 2,3,5,6-tetramethylterephthalic acid (0.020 g, 0.09 mmol) were dissolved in 20 mL of DEF in a glass jar. 5 mg of filtered HKUST-1 crystals were added and well dispersed on the bottom of the glass jar. The glass jar was heated at 75 ºC. After 48 hours, the mother liquor was quickly decanted and HKUST-1@IRMOF-18 crystals were washed with fresh DEF and dichloromethane.

Sequential synthesis of MOF-5 with Co-bdc

$Zn(NO_3)_2 \cdot 6H_2O$ (0.150 g, 0.504 mmol) was dissolved in DEF (10 mL), to which 10 mL of DEF solution containing terephthalic acid (0.0632 g, 0.379 mmol) was added. 15 mg of filtered Co-bdc crystals were placed in a glass jar followed by addition of MOF-5 precursor solution and capped tightly. The glass jar was heated to 100 ºC with ramping rate of 1 ºC/min. After 24 hours, the mother liquor was quickly decanted and Co-BDC@MOF-5 crystals were washed with fresh DEF.

Sequential synthesis of IRMOF-20 with HKUST-1[18]

$Zn(NO_3)_2 \cdot 4H_2O$ (0.113 g, 0.432 mmol) was dissolved in DEF (2.5 mL) and added to a DEF solution (2.5 mL) of thieno[3,2-b]thiophene-2,5-dicarboxylic acid[19,20] (0.0281 g, 0.123 mmol). The mixture was placed in a glass jar and 4 mg of filtered HKUST-1 crystals were added. The

octahedral HKUST-1 crystals were well dispersed on the bottom of the glass jar. The glass jar was heated at 100 °C with ramping rate of 1 °C/min. After 18 hours, the mother liquor was quickly decanted and HKUST-1@IRMOF-20 crystals were washed with fresh DEF.

Preparation of PCN-68@MOF-5

$Zn(NO_3)_2 \cdot 6H_2O$ (0.150 g, 0.504 mmol) was dissolved in DEF (5 mL), to which 5 mL of DEF solution containing terephthalic acid (0.0632 g, 0.379 mmol) was added. 1.5 mg of filtered PCN-68 crystals were placed in a glass jar followed by addition of MOF-5 precursor solution and capped tightly. The glass jar was heated to 85 °C with ramping rate of 1 °C/min. After 24 hours, the mother liquor was quickly decanted and PCN-68@MOF-5 crystals were washed with fresh DEF.

Preparation of UiO-67@HKUST-1[21]

20 mg of UiO-67 were added to a methanol (25 mL) solution with Benzene-1,3,5-tricarboxylic acid (0.0800 g, 0.381 mmol) and sonicated for 90 minutes. 25 mL of methanol solution containing $Cu(NO_3)_2 \cdot 2.5H_2O$ (0.910 g, 3.91 mmol) was added to the solution. The mixture was transferred to a 100 mL vial, sonicated for 10 min, and placed in room temperature for 2 hours. The blue powder was collected by centrifugation and washed 3 times with methanol.

Preparation of UiO-66@MIL-88A(Fe)

20 mg of UiO-66 were added to a 5 mL aqueous solution of Fumaric acid (0.0487 g, 0.422 mmol) and sonicated for 90 minutes. 5 mL of distilled water solution containing $FeCl_3 \cdot 6H_2O$ (0.114 g, 0.422 mmol) was added to the solution. The mixture was transferred to Teflon vessel within the autoclave and heated at 85 °C. After 6 hours, the orange powder was collected by centrifugation and washed 3 times with distilled water.

Preparation of UiO-66@MIL-88B(Fe)

20 mg of UiO-66 were added to a 3 mL DMF solution containing terephthalic acid (0.0553g, 0.333 mmol) and sonicated for 90 minutes. $FeCl_3 \cdot 6H_2O$ (0.0900 g, 0.333 mmol) in 2 mL of DMF solution was fully dissolved by adding 0.4 mL of NaOH solution (0.67M) and added to the solution. The mixture was transferred to Teflon vessel within the autoclave and heated to 100 ºC with heating ramp of 3°C/min. After 12 hours, the orange powder was collected by centrifugation and washed 3 times with distilled water.

Preparation of UiO-66@MIL-88C(Fe)

20 mg of UiO-66 were added to a 5 mL of DMF solution containing 2,6-naphtalene dicarboxylic acid (0.100 g, 0.409 mmol) and sonicated for 90 minutes. 5 mL of DMF solution containing $FeCl_3 \cdot 6H_2O$ (0.128 g, 0.473 mmol) was added to the solution. The mixture was transferred to 250 mL round-bottom flask and heated to 130 ºC with stirring (300 rpm). After 18 hours, the orange powder was collected by centrifugation and washed 3 times with DMF.

Preparation of UiO-67@MIL-88C(Fe)

20 mg of UiO-67 were added to a 5 mL of DMF solution containing 2,6-naphtalene dicarboxylic acid (0.100 g, 0.409 mmol) and sonicated for 90 minutes. 5 mL of DMF solution containing $FeCl_3 \cdot 6H_2O$ (0.128 g, 0.473 mmol) was added to the solution. The mixture was transferred to 250 mL round-bottom flask and heated to 130 ºC with stirring (300 rpm). After 18 hours, the orange powder was collected by centrifugation and washed 3 times with DMF.

Supplementary Figures

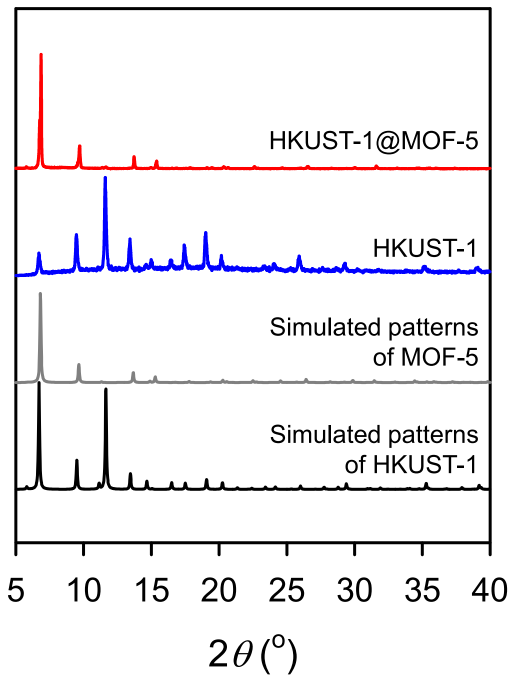

Supplementary Figure 12 | XRPD patterns of HKUST-1(blue) and HKUST-1@MOF-5 (red) with the simulated XRPD patterns from single crystal data from HKUST-1 (black) and MOF-5 (gray).

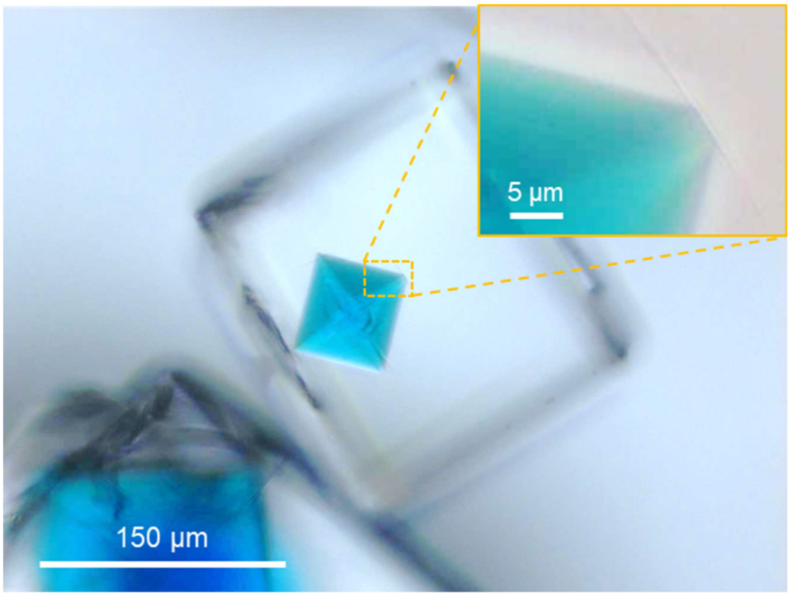

Supplementary Figure 13| Optical microscope image of HKUST-1@MOF-5 crystals. Inset image is high magnification optical microscope image at the interface of a HKUST-1@MOF crystal which clearly indicates the seamless interface between the HKUST-1 and MOF-5 crystals.

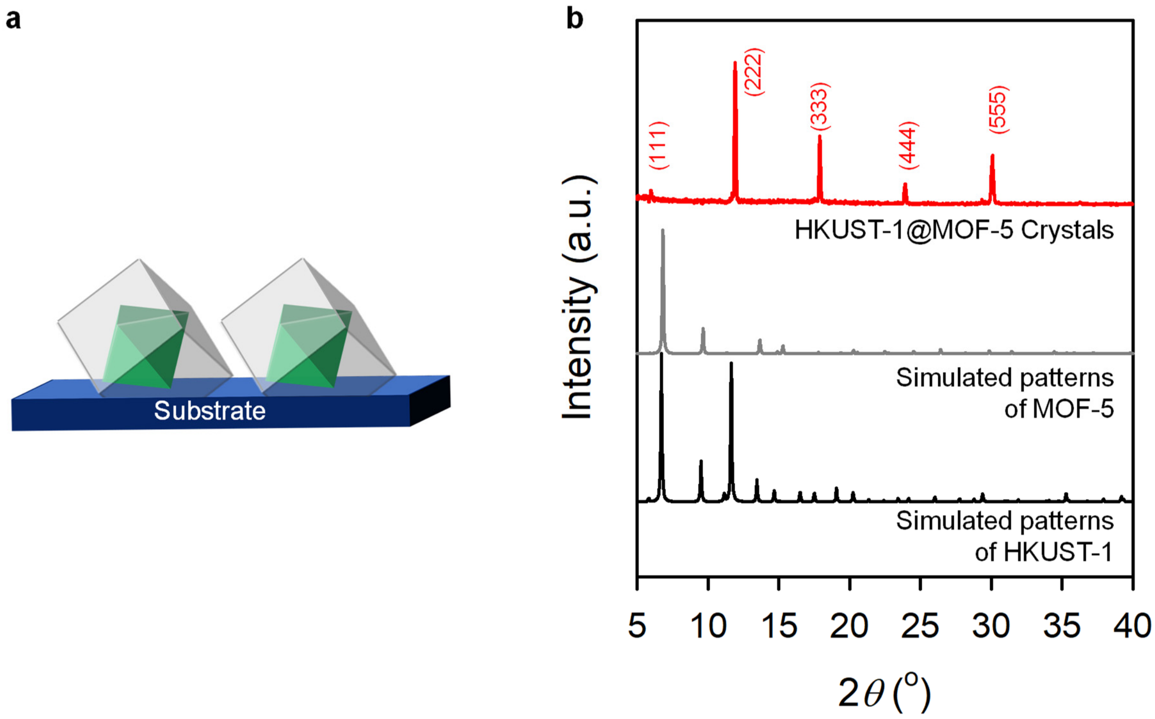

Supplementary Figure 14 | a, Schematic image of HKUST-1@MOF-5 crystals on the substrate for X-ray powder diffraction measurements. **b**, XRPD patterns of HKUST-1@MOF-5 crystals (red) placed on the substrate as illustrated in Supplementary Figure 14a with the simulated XRPD patterns from single crystal data from HKUST-1 (black) and MOF-5 (gray). The XRPD patterns of HKUST-1@MOF-5 crystals showed strong (111), (222), (333), (444), and (555) diffraction peaks, unambiguously confirm the facet-aligned formation of HKUST-1@MOF-5 composites.

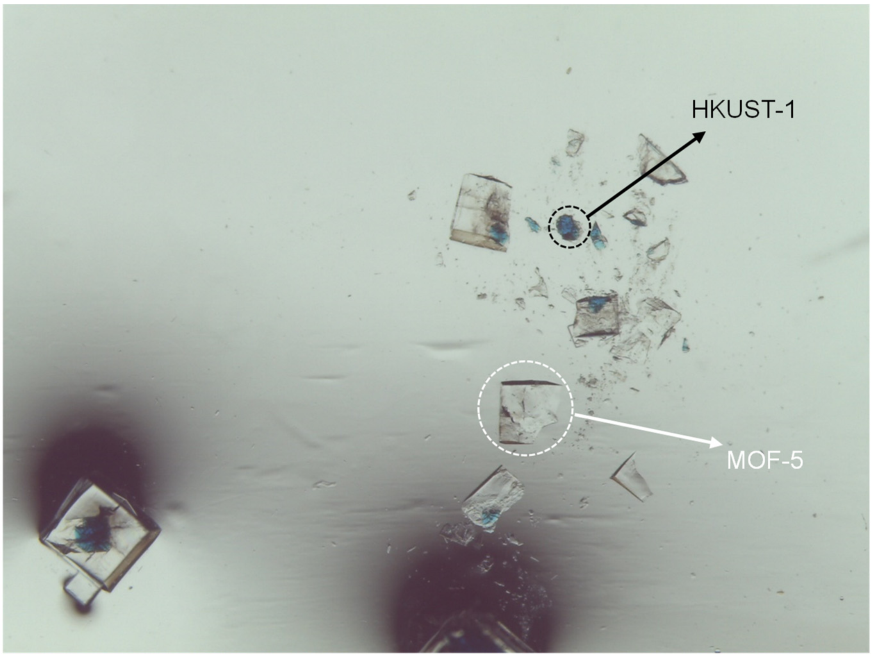

Supplementary Figure 15 | Optical microscope image obtained by breaking the HKUST-1@MOF-5 crystal. Blue crystal in black dotted circle and colorless crystal in white dotted circles were HKUST-1 and MOF-5, respectively, which were confirmed by single-crystal X-ray diffraction (SCD) analysis. The single-crystal X-ray diffraction data were measured with transmission-mode as Debye-Scherrer Pattern on a Rayonix MX225HS CCD detector at BL2D SMC with a silicon (111) double crystal monochromator (DCM) at the Pohang Accelerator Laboratory, Republic of Korea.

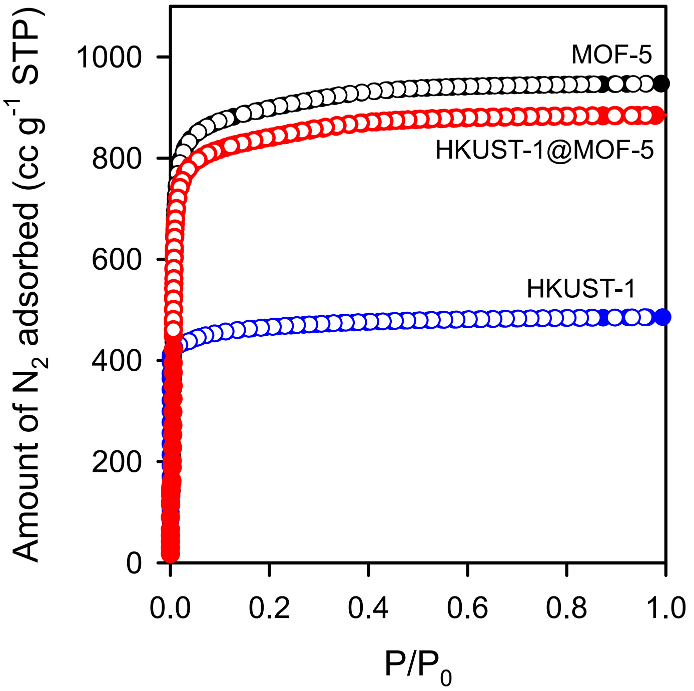

Supplementary Figure 16 | N_2 sorption isotherm measurements at 77 K of HKUST-1 (blue), MOF-5 (black), and HKUST-1@MOF-5 (red). Filled and open symbols correspond to adsorption and desorption, respectively).

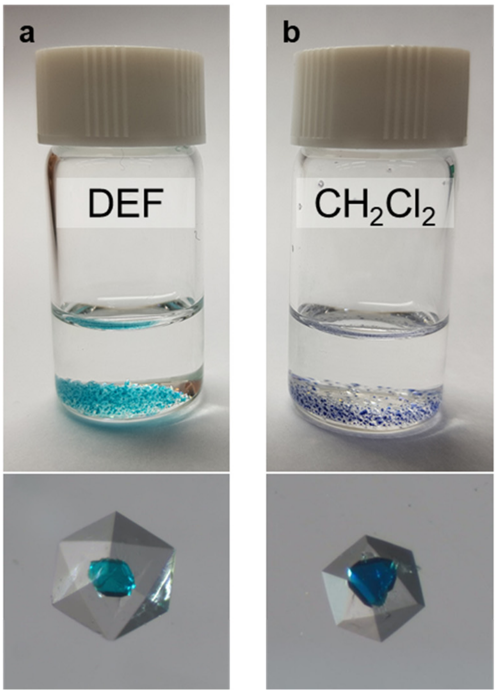

Supplementary Figure 17 | **a,** Photographs images and corresponding optical microscope images of HKUST-1@MOF-5 crystals in diethylformamide (DEF). **b,** Photographs images and corresponding optical microscope images of HKUST-1@MOF-5 crystals in dichloromethane (CH_2Cl_2). Strong color change of HKUST-1 in HKUST-1@MOF-5 crystals depending on solvents indicates the accessible and interconnected pore space at the interface between HKUST-1 and MOF-5.

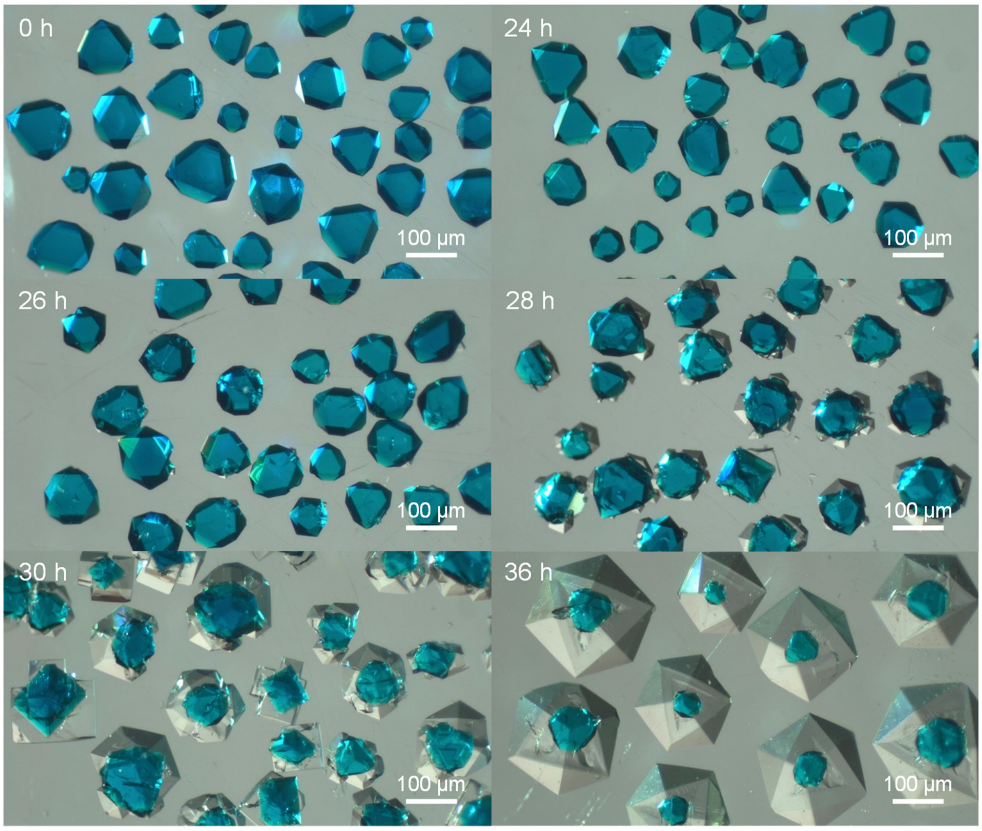

Supplementary Figure 18 | Optical microscope images monitoring the growth process of single crystal HKUST-1@MOF-5 from octahedral HKUST-1 at different time scales.

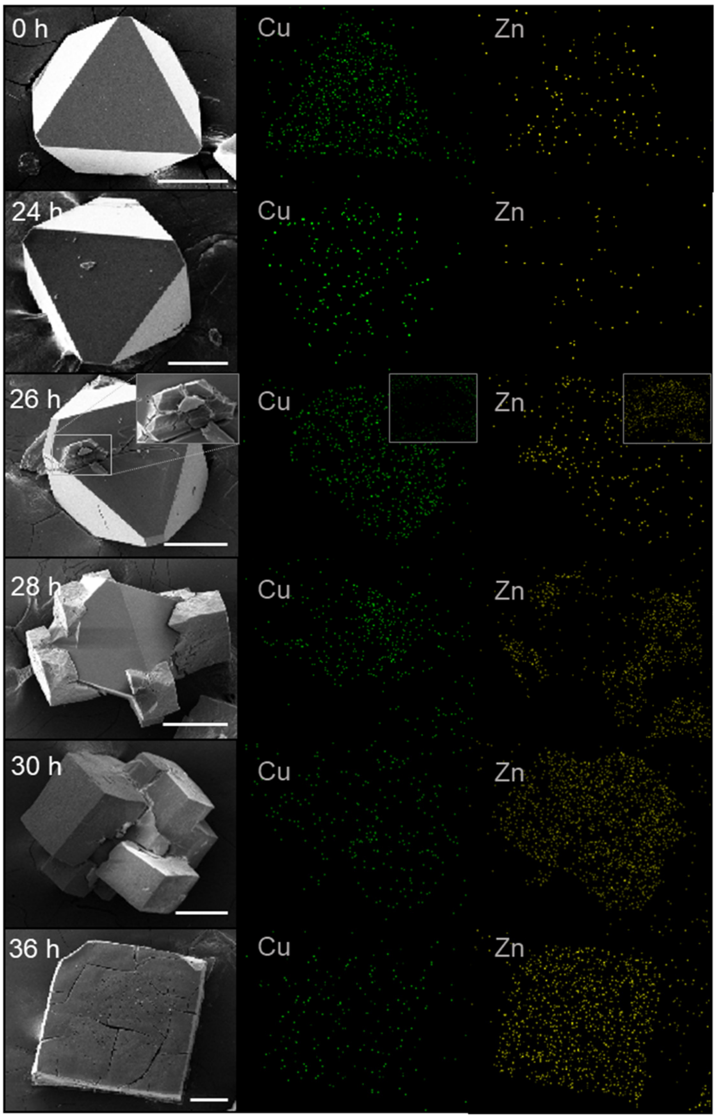

Supplementary Figure 19 | Scanning electron microscopy (SEM) images along with the corresponding energy dispersive X-ray spectroscopy (EDS) elemental maps monitoring the growth process of single crystal HKUST-1@MOF-5 from octahedral HKUST-1 at different time scales. Scale bar : 100 μm.

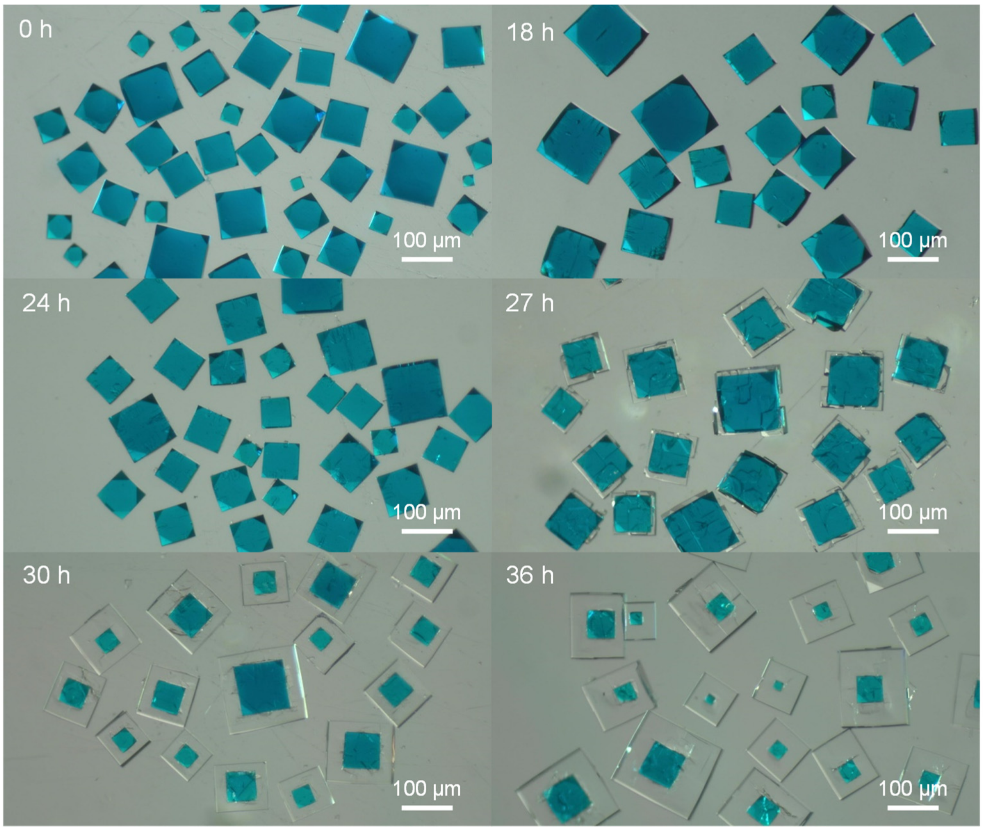

Supplementary Figure 20 | Optical microscope images monitoring the growth process of single crystal HKUST-1@MOF-5 from cubic HKUST-1 at different time scales.

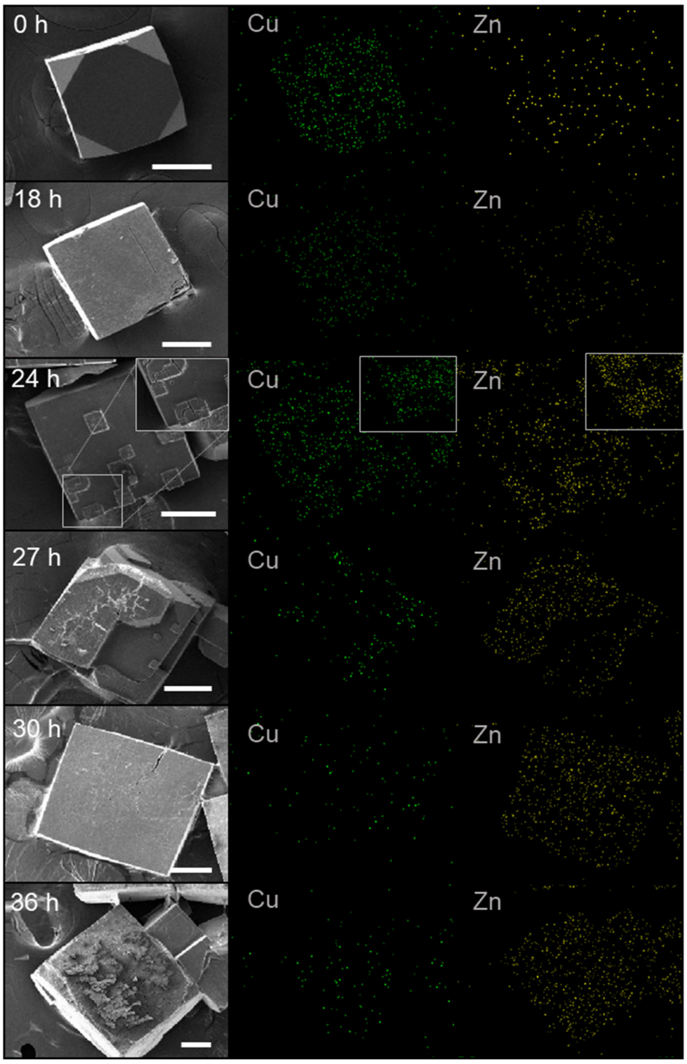

Supplementary Figure 21 | SEM images along with the corresponding EDS elemental maps monitoring the growth process of single crystal HKUST-1@MOF-5 from cubic HKUST-1 at different time scales. Scale bar : 100 μm.

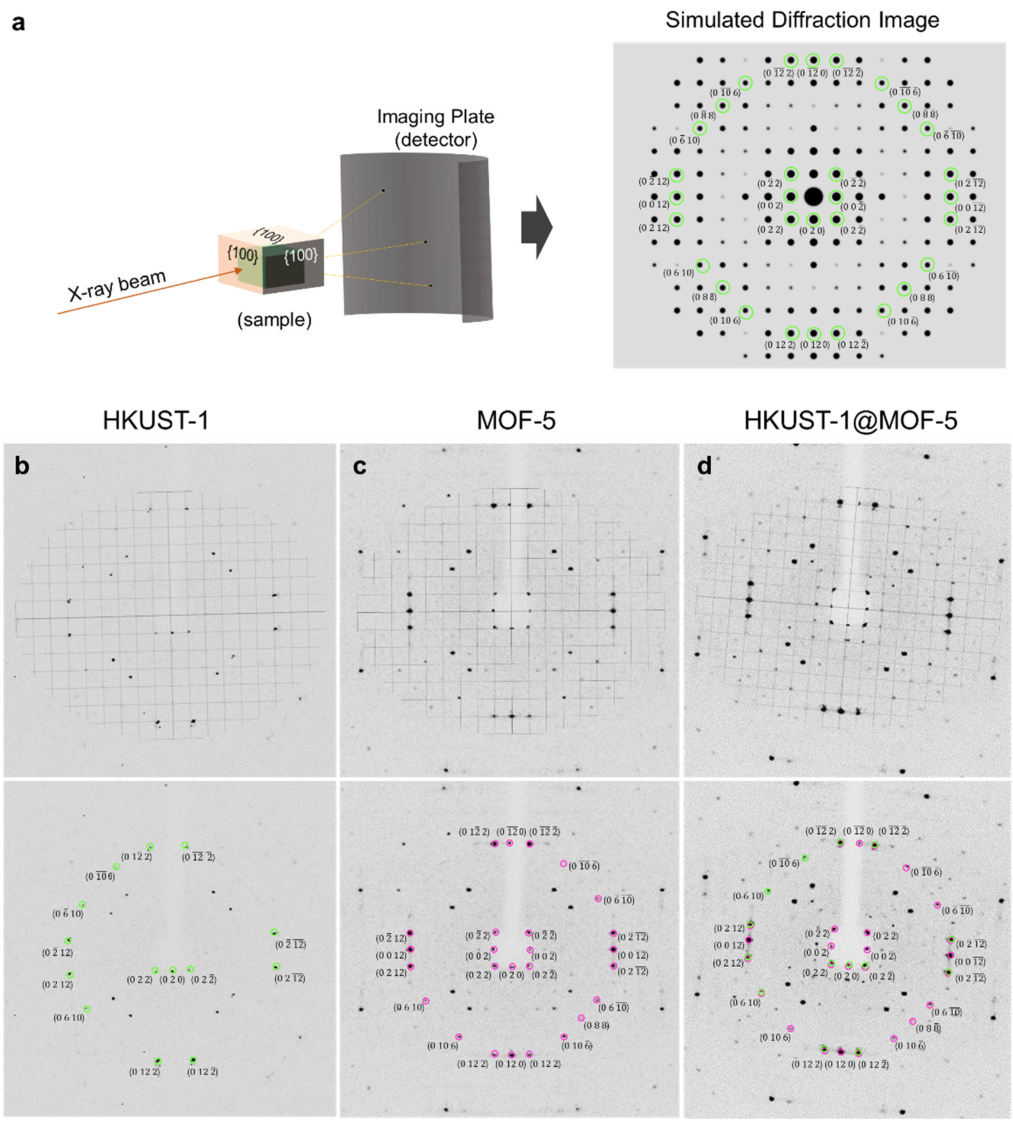

Supplementary Figure 22 | a, Schematic illustration of the measurement of the diffraction images and simulated X-ray diffraction image. X-ray diffraction images for cubic shape HKUST-1, MOF-5 and HKUST-1@MOF-5, which were obtained by incident X-ray beam perpendicular to the {100} plane with 1° of ω–axis oscillation. **b,** HKUST-1. **c,** MOF-5. **d,** HKUST-1@MOF-5. Note that the (h k l) of diffraction peaks assigned when the X-ray beam is incident along the [100] direction. A simulated X-ray diffraction image was generated under the condition that the incident X-ray beam is perpendicular to the (100) plane of MOF-5 using CrystalMaker SingleCrystal

software (version 3.1.4, CrystalMaker Software Ltd, Oxford, England).

The diffraction images are 4-fold symmetric with characteristic reflections of {0 2 0}, {0 2 2}, {0 2 12}, {0 12 0}, and {0 6 10}, which implies that they all are well-aligned to the [100] direction along the X-ray beam. Thus, the results have demonstrated that the cubic HKUST-1@MOF-5 is built from the epitaxial growth. Note that the X-ray beam size (500 μm x 500 μm) is large enough to cover the whole sample, where the diffraction image of the core-shell must include the reflections of both the crystals, core HKUST-1 and shell MOF-5.

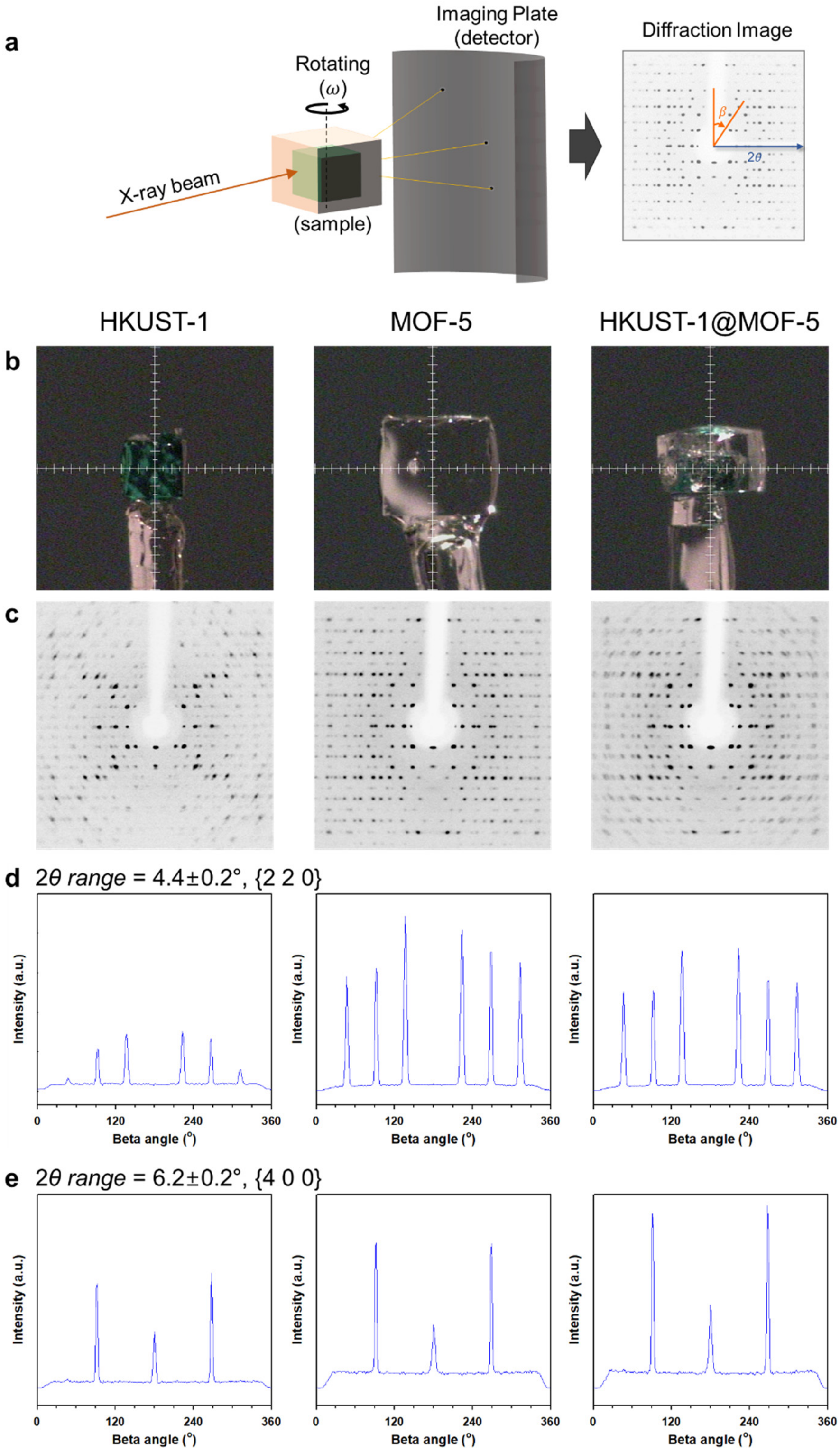

Supplementary Figure 23 | a, Schematic illustration of the measurement of the diffraction images rotating 180° on the ω–axis and X-ray diffraction image. **b,** Crystal images of HKUST-1, MOF-5, and HKUST-1@MOF-5 before the measurement. **c**, Corresponding diffraction

images measured while rotating 180° on the ω–axis. **d**, Intensity profiles obtained by the intensity integration per the β angle within the same 2θ range of 4.4±0.2°, i.e. {h k l} = {2 2 0}. **e**, Intensity profiles obtained by the intensity integration per the β angle within the same 2θ range of 6.2±0.2°, i.e. {h k l} = {4 0 0}.

For the HKUST-1@MOF-5, the periodic peaks without any splitting indicate that the core crystal and shell crystal are arrayed along the ω–axis.

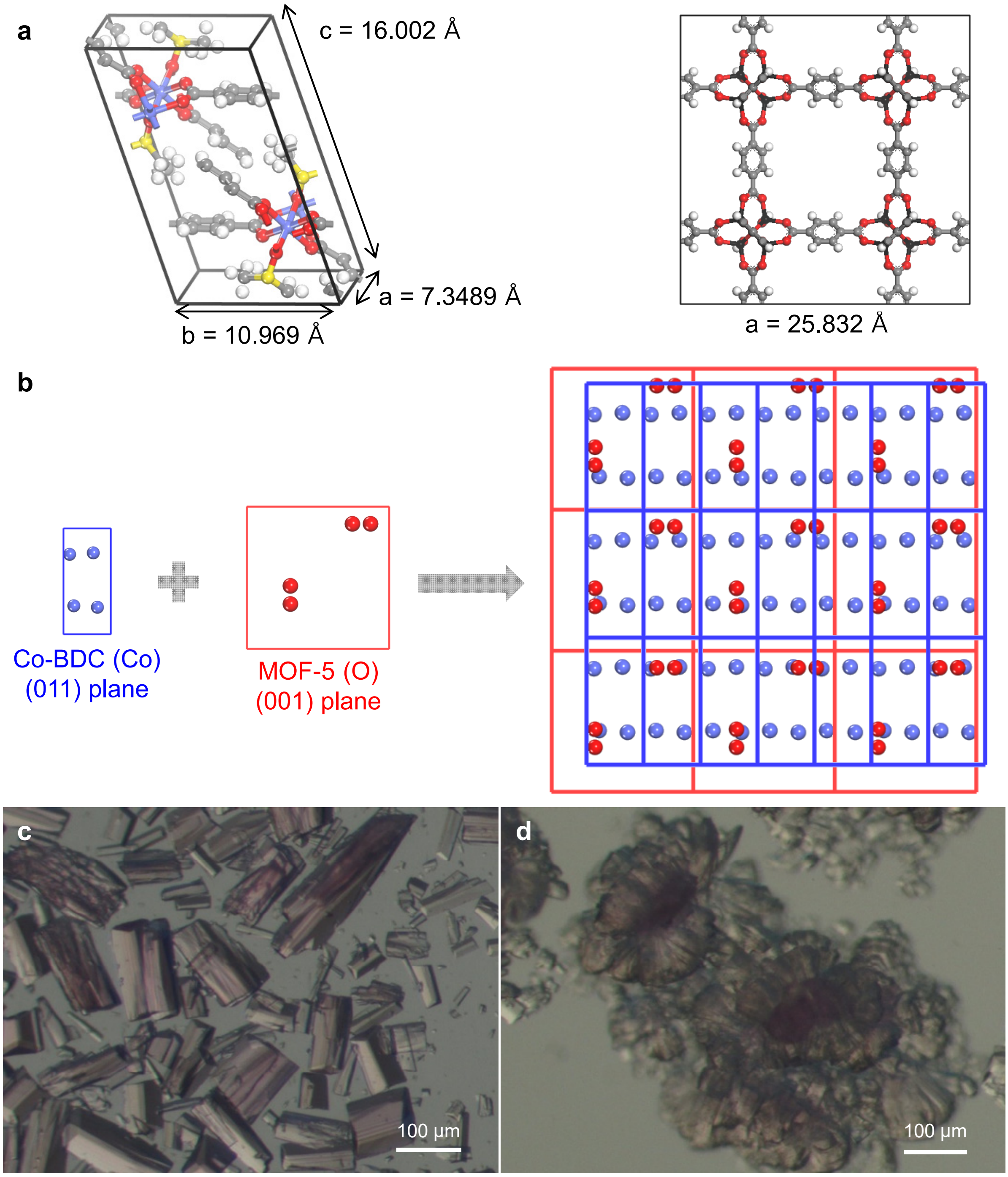

Supplementary Figure 24 | a, The unit cell of Co-bdc (Triclinic $P\bar{1}$, a=7.3489 Å, b=10.969 Å, c=16.002 Å, α=72.51°, β=78.52°, and γ=73.12°, LOMQOV) and MOF-5 (Cubic $Fm\bar{3}m$, a = b = c = 25.832 Å, α = β = γ = 90°, EDUSIF). These two structures have totally different symmetry system with different lattice parameters **b,** Result position of surface pattern matching between (011) plane of Co-bdc and (001) plane of MOF-5. The chemical

connection points cannot be well-matched with each other. If the shell MOF (MOF-5) grow on different sites, there is a possibility that the shell MOFs cannot meet with single crystal and it will lead polycrystalline shape. **c**, Optical microscope images of rod shaped Co-bdc crystal. **d**, Optical microscope image of Co-bdc/MOF-5.

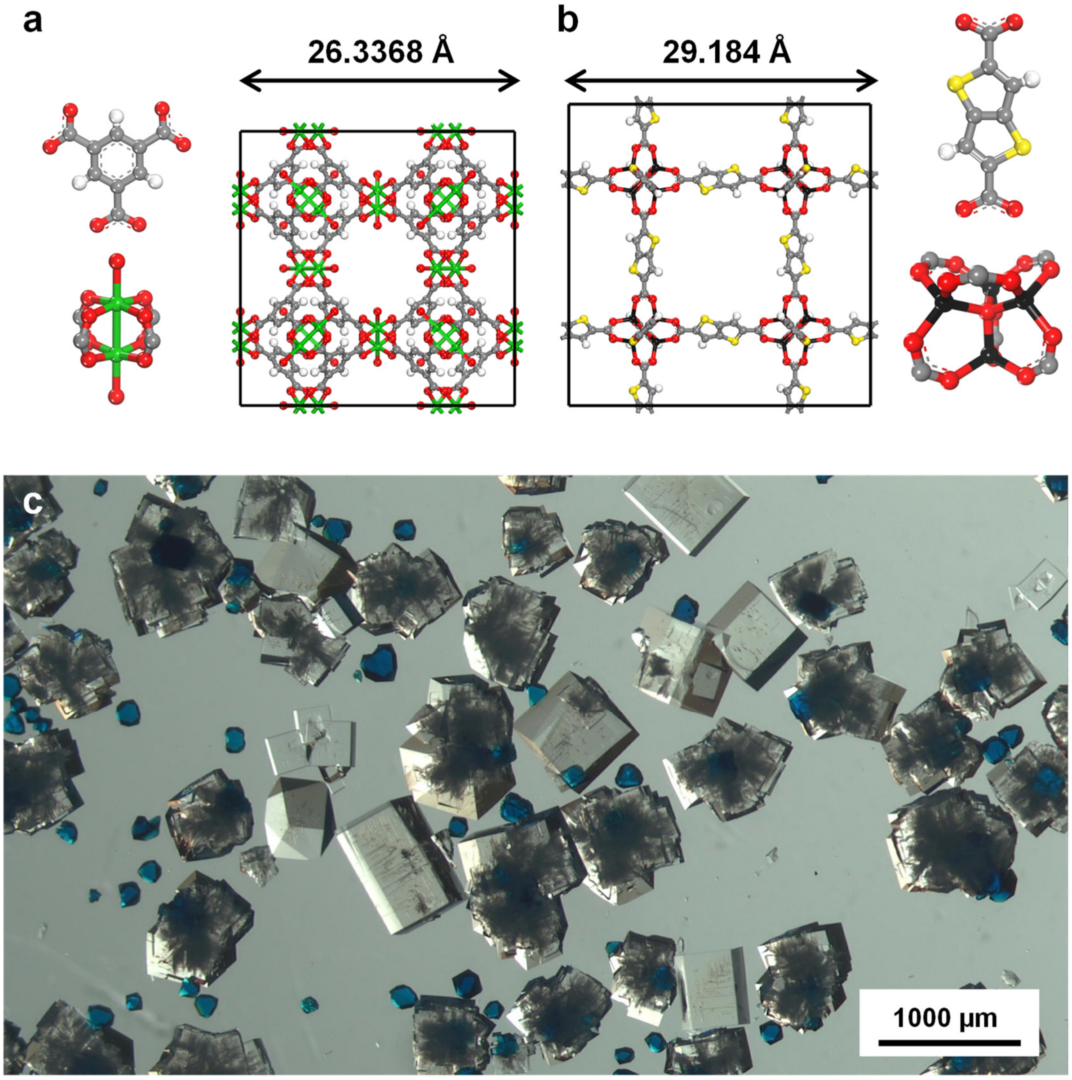

Supplementary Figure 25 | a, Components of HKUST-1, btc linker and Cu paddlewheel cluster, and HKUST-1 structure (cubic $Fm\bar{3}m$ a = 26.3368 Å, α = β = γ = 90˚, XAMDUM). **b**, Components of IRMOF-20, thieno[3,2-b]thiophene-2,5-dicarboxylic acid linker and Zn_4O cluster, and IRMOF-20 structure (cubic $Fm\bar{3}m$ a = 29.184 Å, α = β = γ = 90˚, VEBHUG). **c**, Optical microscope image of HKUST-1/ IRMOF-20.

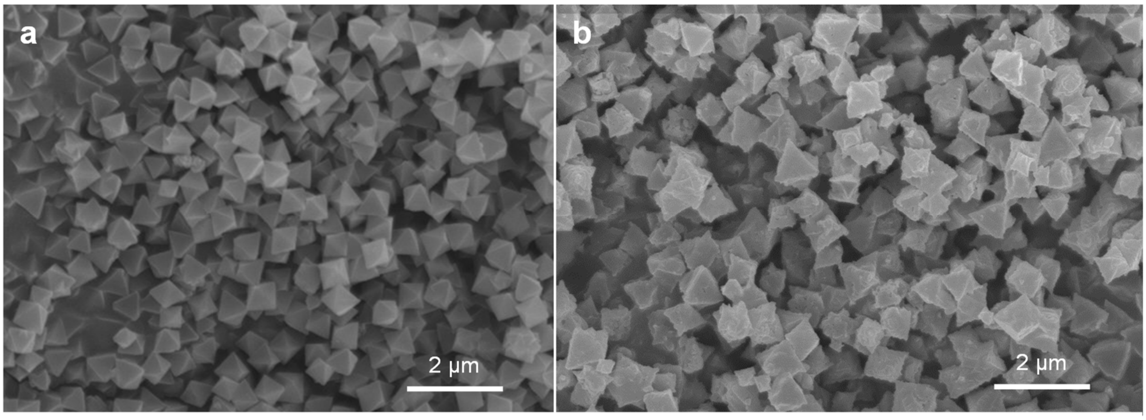

Supplementary Figure 26 | SEM image of **a**, UiO-67 and **b**, UiO-67@HKUST-1.

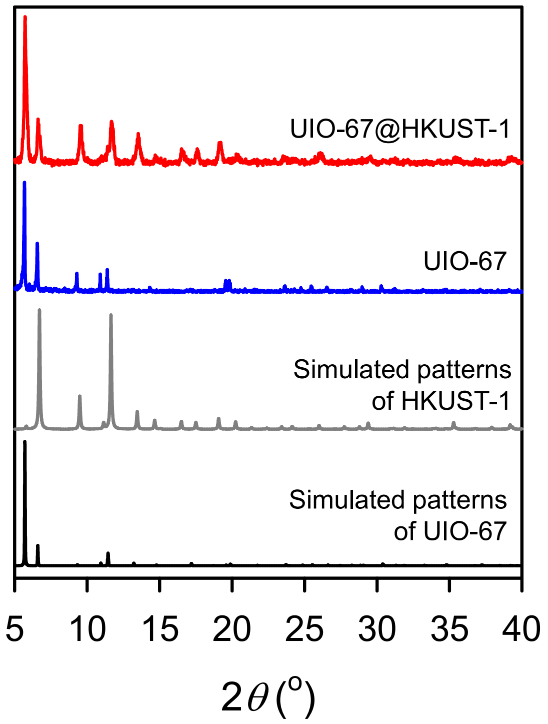

Supplementary Figure 27 | XRPD patterns of UiO-67(blue) and UiO-67@HKUST-1 (red) with the simulated XRPD patterns from single crystal data from UiO-67 (black) and HKUST-1 (gray).

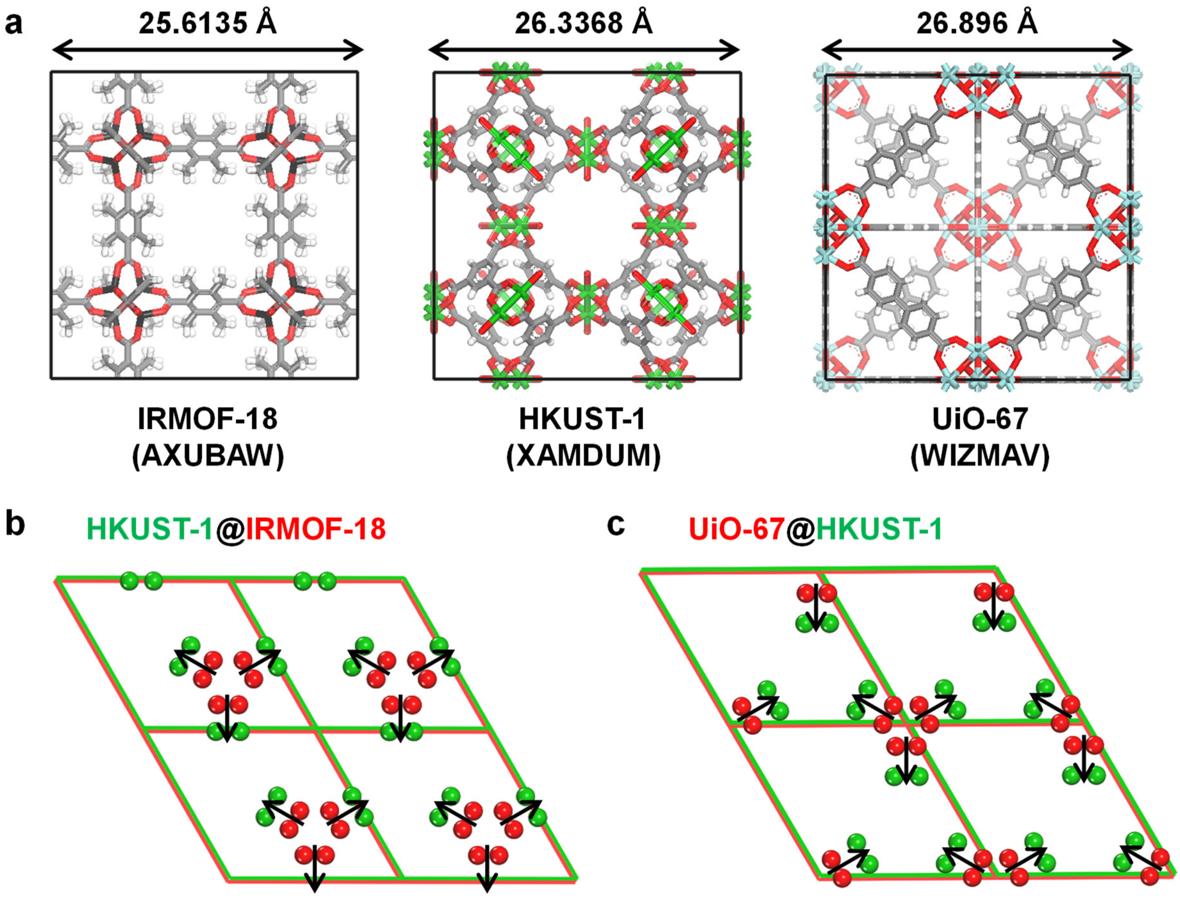

Supplementary Figure 28 | a, The unit cells of IRMOF-18(left), HKUST-1(middle) and UiO-67(right). They have same symmetry (cubic $Fm\bar{3}m$) and similar lattice parameters (25. 6135 Å, 26.3368 Å and 26.896 Å for IRMOF-18, HKUST-1 and UiO-67 respectively). It leads to similar 2D lattice parameters of chemical connection points from each structure. **b**, Result of chemical connection points matching of (111) plane of HKUST-1/IRMOF-18. Green and red spheres represent copper and oxygen atoms, respectively. Green and red line represents unit cell of HKUST-1 and IRMOF-18, respectively. **c**, Results of chemical connection points matching of (111) plane of UiO-67/HKUST-1. Green and red spheres represent copper and oxygen atoms, respectively. Red and green line represents unit cell of UiO-67 and HKUST-1.

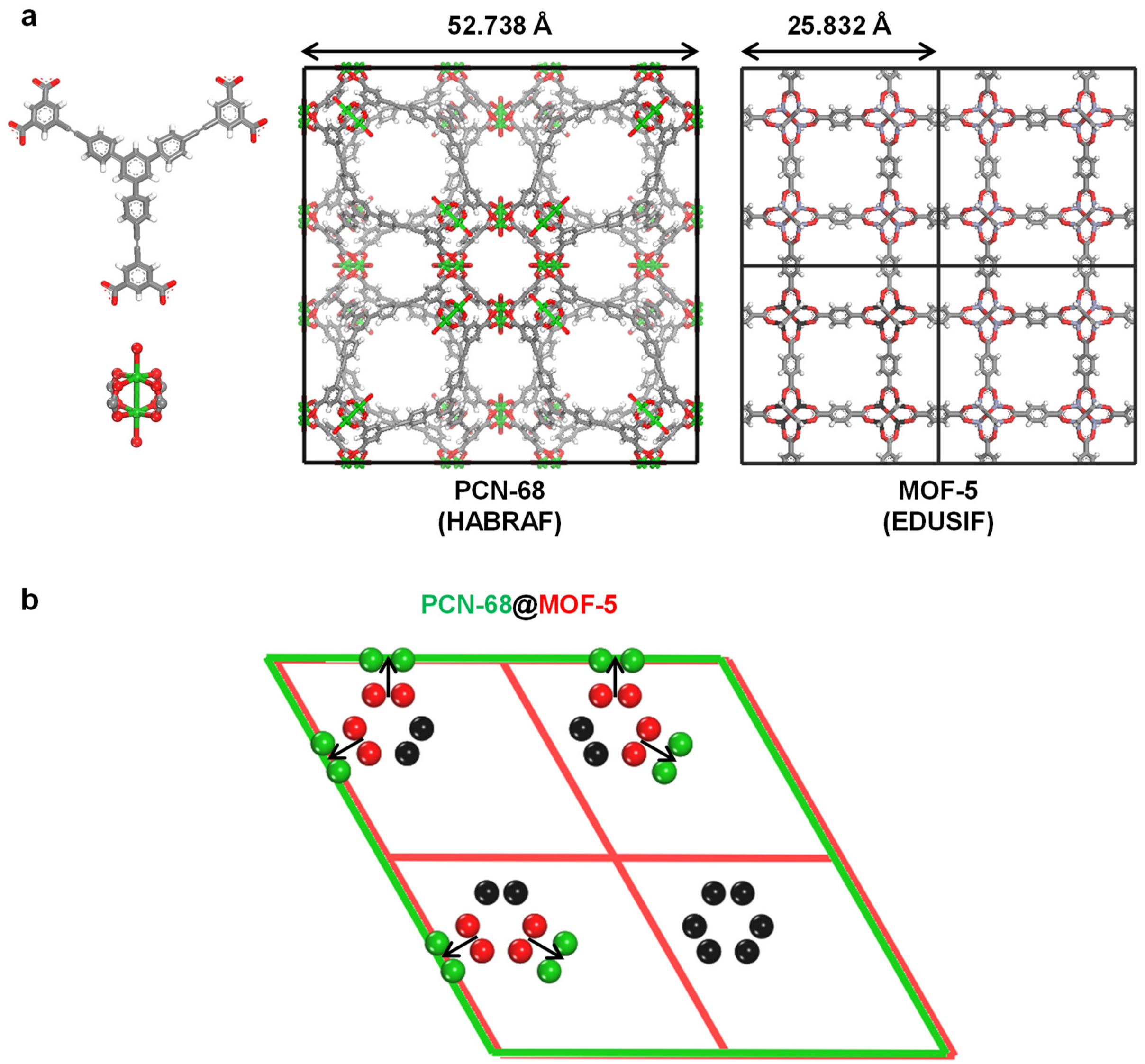

Supplementary Figure 29 | a, The components of PCN-68 (left), the unit cells of PCN-68 (middle) and MOF-5 (right). PCN-68 and MOF-5 have same symmetry (cubic $Fm\bar{3}m$) have similar supercell lattice parameters. **b,** Result of chemical connection points matching of (111) plane of PCN-68/MOF-5. The green line represents 2D lattice of chemical connection points from PCN-68 and the red line represents that of MOF-5. MOF-5 has twice as large chemical connection points per unit area than that of PCN-68. Green, red, and black spheres represent copper, oxygen, and dangling oxygen atoms, respectively.

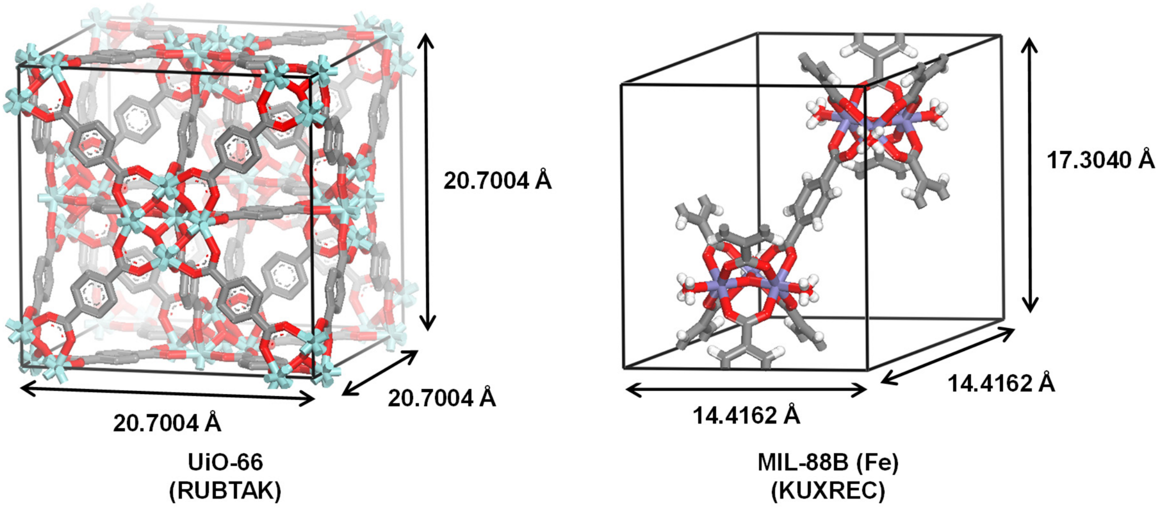

Supplementary Figure 30 | The structures of UiO-66 (left) and MIL-88B(Fe) (right). UiO-66 is cubic crystal system ($Fm\overline{3}m$, a = 20.7004 Å, α = β = γ = 90˚, RUBTAK) and MIL-88B(Fe) is hexagonal crystal system ($P6_3/mmc$, a = b = 14.4162 Å and c = 17.3040 Å, α = β = 90˚, γ = 120˚, KUXREC).

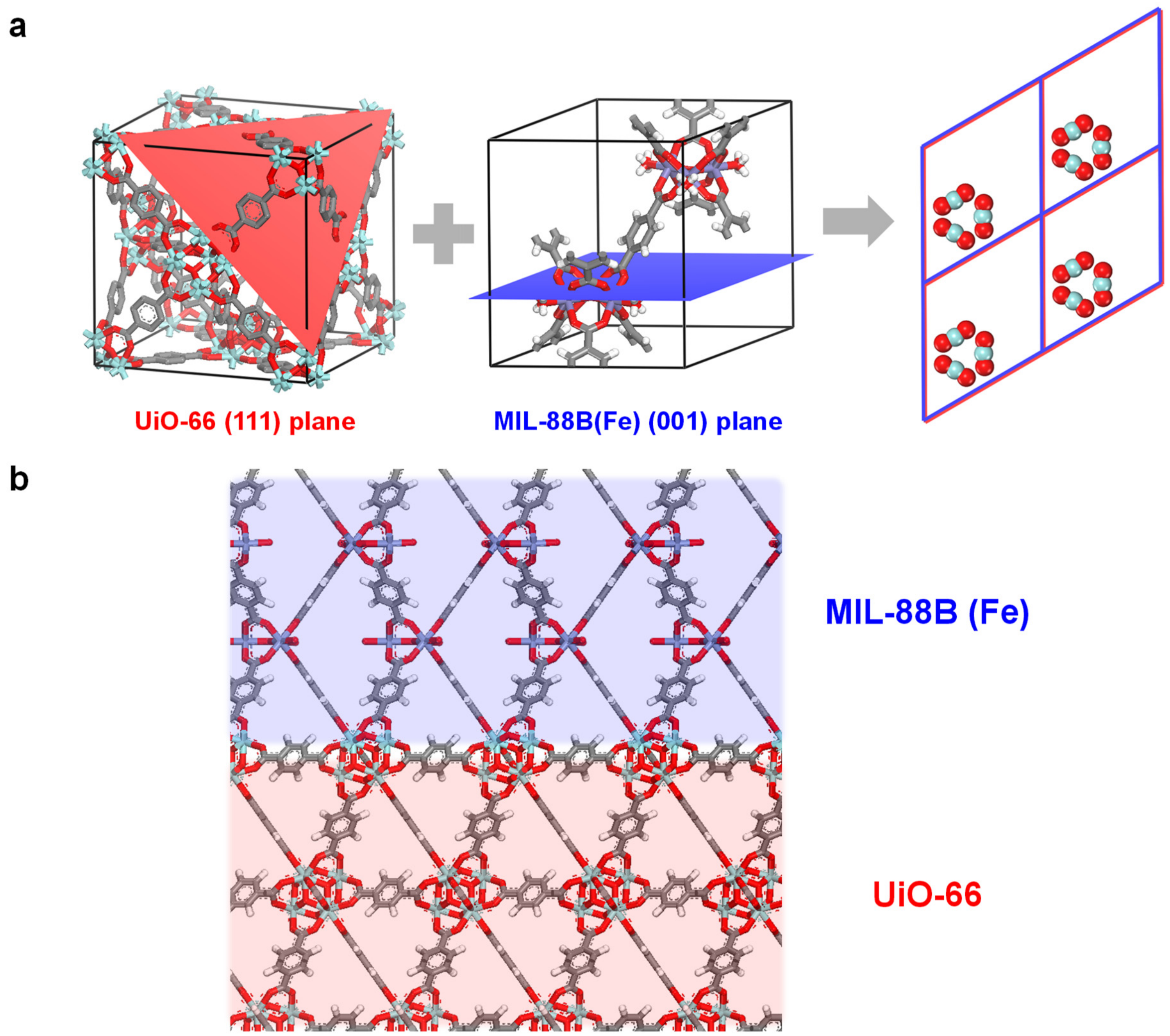

Supplementary Figure 31 | Chemical connection points matching and computational structural model of UiO-66@MIL-88B(Fe). **a,** Chemical connection points matching between (111) plane of UiO-66 and (001) plane of MIL-88B (Fe). **b,** Computational structural model from chemical connection points of UiO-66/MIL-88B(Fe) system.

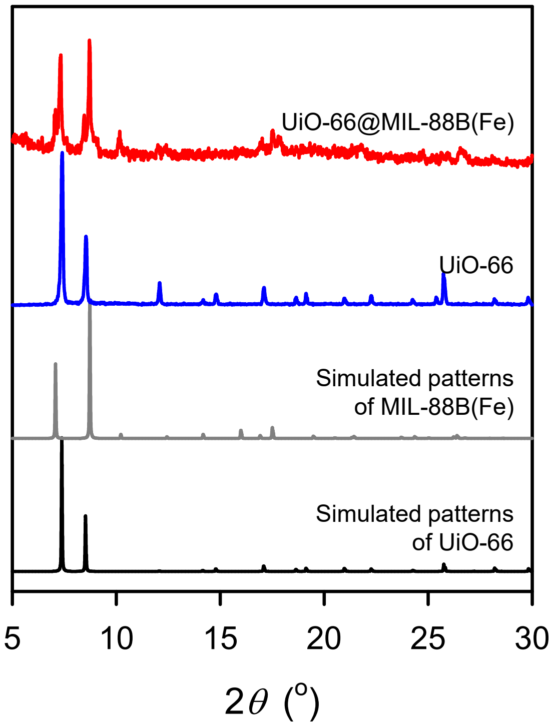

Supplementary Figure 32 | XRPD patterns of UiO-66(blue) and UiO-66@MIL-88B(Fe) (red) with the simulated XRPD patterns from single crystal data from UiO-66 (black) and MIL-88B(Fe) (gray).

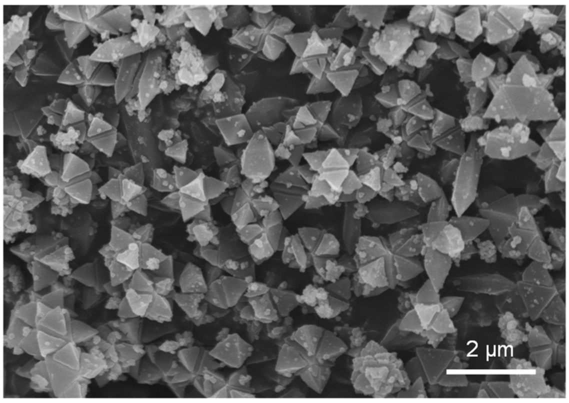

Supplementary Figure 33 | SEM image of UiO-66@MIL-88B(Fe) after reaction for 12 hours.

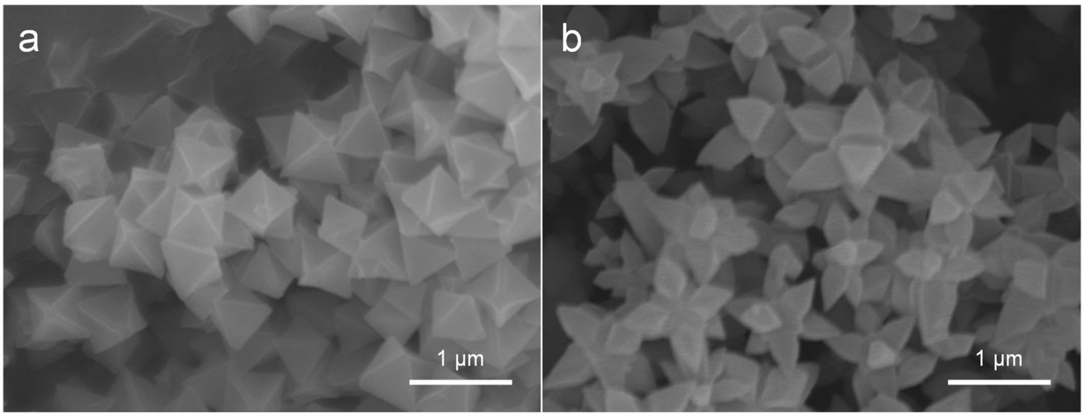

Supplementary Figure 34 | SEM images of **a**, UiO-67 and **b**, UiO-67@MIL-88C. SEM image of UiO-67@MIL-88C clearly showed newly grown triangular pyramid crystals on the each {111} surface of octahedral UiO-67 crystals, indicating successful synthesis of MOF@MOF structures.

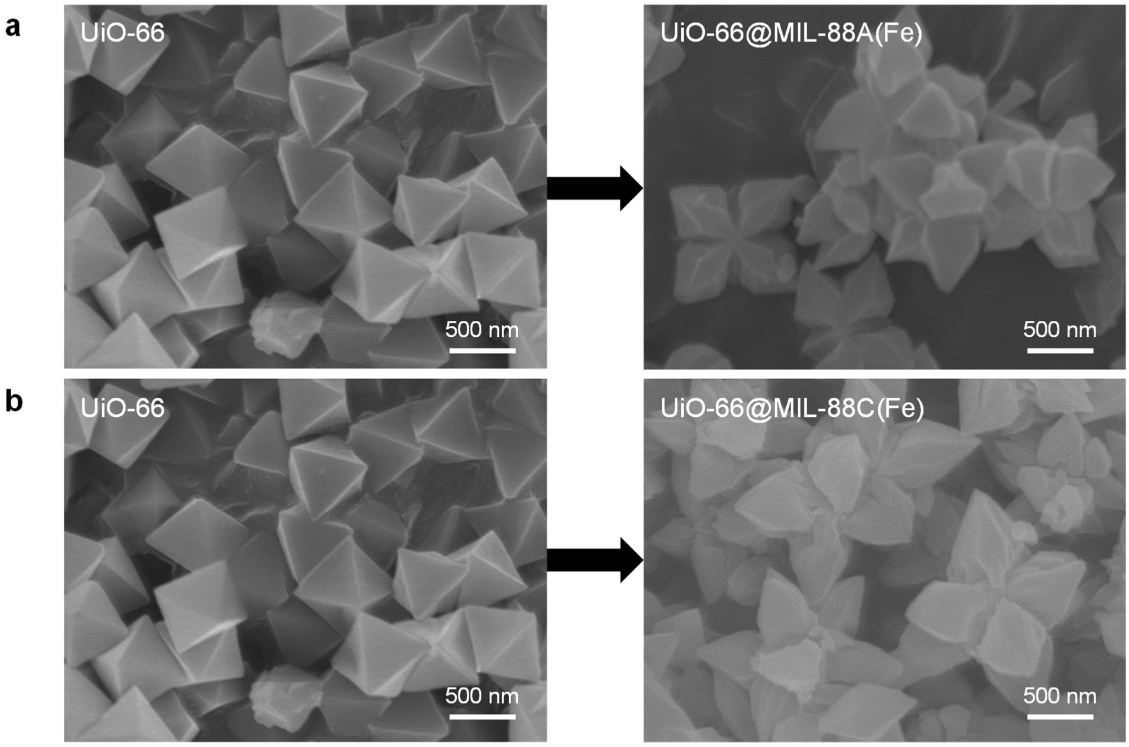

Supplementary Figure 35 | SEM images of **a**, UiO-66 and UiO-66@MIL-88A(Fe) and **b**, UiO-66 and UiO-66@MIL-88C(Fe). Both of SEM images of UiO-66@MIL-88A(Fe) and UiO-66@MIL-88C(Fe) clearly showed newly grown triangular pyramid crystals on the each {111} surface of octahedral UiO-66 crystals, indicating successful synthesis of MOF@MOF structures.

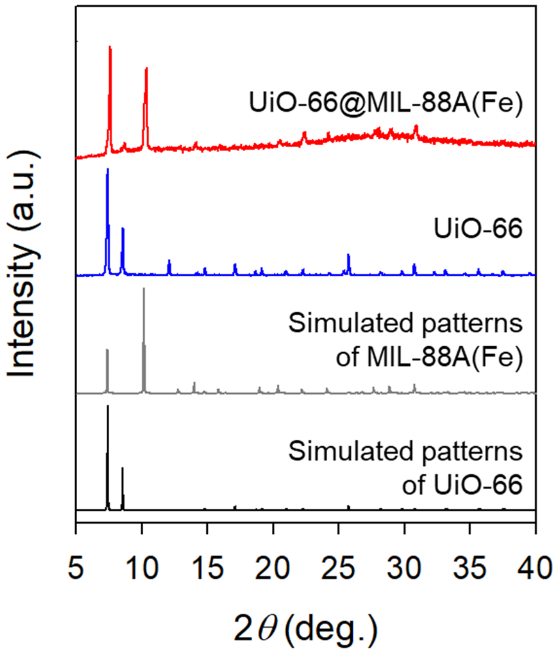

Supplementary Figure 36 | XRPD patterns of UiO-66(blue) and UiO-66@MIL-88A(Fe) (red) with the simulated XRPD patterns from single crystal data from UiO-66 (black) and MIL-88A(Fe) (gray).

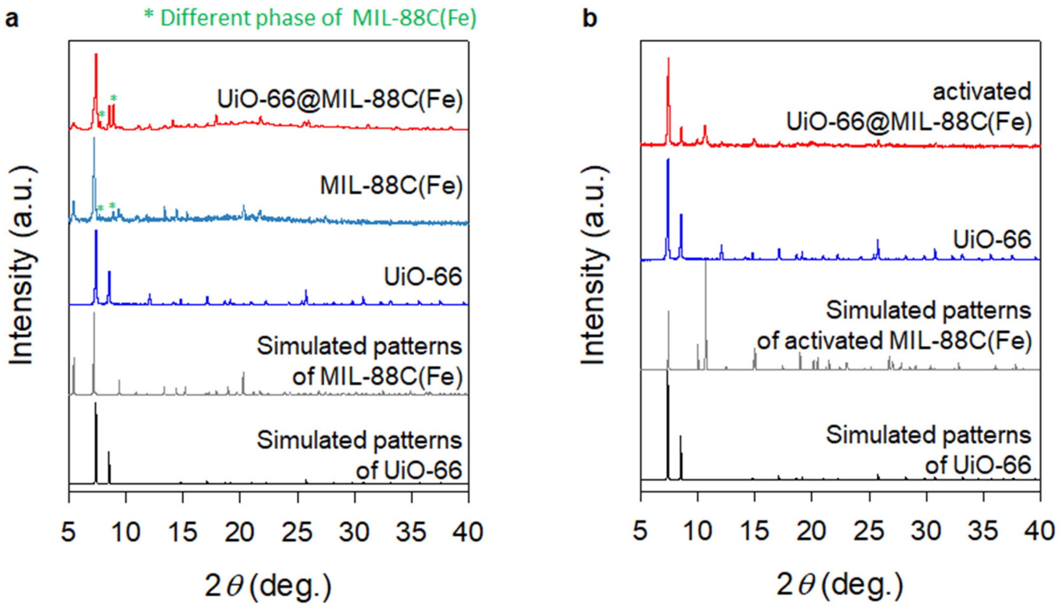

Supplementary Figure 37 | a, XRPD patterns of UiO-66(blue), MIL-88C(Fe) (light blue), and UiO-66@MIL-88C(Fe) (red) with the simulated XRPD patterns from single crystal data from UiO-66 (black) and MIL-88C(Fe) (gray). Note that the peaks marked with an asterisk result from a different phase of MIL-88C(Fe).[22] **b,** XRPD patterns of UiO-66 (blue) and activated UiO-66@MIL-88C(Fe) (red) with the simulated XRPD patterns from single crystal data form UiO-66 (black) and activated MIL-88C(Fe) (gray), which indicate that the MIL-88C(Fe) grown on UiO-66 is the pure phase.

Supplementary Table 3. The lattice parameters of {001} of MIL-88 series in open and dry phase taken from supplementary reference 22

Phase	***MIL-88A***	***MIL-88B***	***MIL-88C***
dry	7.7798(10) Å	9.600(1) Å	9.900(1) Å
open	13.8711(10) Å	15.6261(10) Å	18.7529(10) Å

Supplementary References

1. Moghadam, P. Z. et al. Development of a cambridge structural database subset: a collection of metal–organic frameworks for past, present, and future. *Chem. Mater.* **29,** 2618–2625 (2017).

2. Bruno, I. J. et al. New software for searching the Cambridge Structural Database and visualizing crystal structures research papers. *Acta Crystallogr. B* **58,** 389–397 (2002).

3. Dassault Systèmes BIOVIA, Materials Studio, Release 2019.

4. Kwon, O., Park, S., Zhou, H. C. & Kim, J. Computational prediction of hetero-interpenetration in metal-organic frameworks. *Chem. Commun.* **53,** 1953–1956 (2017).

5. Lukose, B. et al. Nanoporous designer solids with huge lattice constant

gradients: Multiheteroepitaxy of metal–organic frameworks. *Nano Lett.* **14,** 1526–1529 (2014).

6. Tovar, T. M. et al. Diffusion of CO_2 in large crystals of Cu-BTC MOF. *J. Am. Chem. Soc.* **138,** 11449-11452 (2016).

7. Lee, K. J. et al. Preparation of Co_3O_4 electrode materials with different microstructures via pseudomorphic conversion of Co-based metal-organic frameworks. *J. Mater. Chem. A.* **35,** 14393–14400 (2014).

8. Liang, W. et al. Site isolation leads to stable photocatalytic reduction of CO_2 over a Rhenium-based catalyst. *chem. Eur. J.* **21,** 18576–18579 (2015).

9. Aujard, I. et al. Tetrahedral onsager crosses for solubility improvement and crystallization bypass. *J. Am. Chem. Soc.* **123,** 8177–8188 (2001).

10. Yuan, D., Zhao, D., Sun, D. & Zhou, H. C. An isoreticular series of metal-organic frameworks with dendritic hexacarboxylate ligands and exceptionally high gas-uptake capacity. *Angew. Chem. Int. Ed.* **49,** 5357–5361 (2010).

11. Yan, Y. et al. Metal-organic polyhedral frameworks : high H_2 adsorption capacities and neutron powder diffraction studies. *J. Am. Chem. Soc.* **132,** 4092–4094 (2010).

12. Zhao, Y., Zhang, Q., Li, Y., Zhang, R. & Lu, G. Large-scale synthesis of monodisperse UiO-66 crystals with tunable sizes and missing linker defects via acid/base co-modulation. *ACS Appl. Mater. Interfaces* **9,** 15079–15085 (2017).

13. Park, J. H., Choi, K. M., Lee, D. K., Moon, B. C. & Shin, S. R. Encapsulation of redox polysulphides via chemical interaction with nitrogen atoms in the organic linkers of metal-organic framework nanocrystals. *Sci. Rep.* **6,** 25555 (2016).

14. Wang, J.et al. Metal-organic frameworks MIL-88A with suitable synthesis conditions and optimal dosage for effective catalytic degradation of Orange G through persulfate activation. *RSC Adv*. **6,** 112502–112511 (2016).

15. Wei, Y. et al. Coordination templated [2+2+2] cyclotrimerization in a porous coordination framework. *Nat. Commun.* **6,** 8348 (2015)

16. Qian, Y. et al. Fe/Fe_3C/N-doped carbon materials from metal–organic framework composites as highly efficient oxygen reduction reaction electrocatalysts. *ChemPlusChem* **81,** 718-723 (2016).

17. Rowsell, J. L. C., Millward, A. R., Park, K. S. & Yaghi, O. M. Hydrogen sorption in functionalized metal-organic frameworks. *J. Am. Chem. Soc.* **126,** 5666–5667 (2004).

18. Rowsell, J. L. C. & Yaghi, O. M. Effects of functionalization, catenation , and variation of the metal oxide and organic linking units on the low-pressure hydrogen adsorption properties of metal-organic frameworks. *J. Am. Chem. Soc* **128,** 1304–1315 (2006).

19. Bugge, A. Metalation of thieno[2,3-b]thiophene and thieno[3,2-b]thiophene with butyllithium. *Acta Chem. Scand.* **22,** 63–69 (1968)

20. Fuller, L. S., Iddon, B. & Smith, K. A. Thienothiophene. Part 2. Synthesis, metalation and bromine→ lithium exchange reactions of thieno [3,2-b] thiophene and its polybromo derivatives. *J. Chem. Soc, Perkin Trans.* **1,** 3465–3470 (1997).

21. Wang, Y. et al. Synthesis of porous Cu_2O/CuO cages using Cu-based metal–organic frameworks as templates and their gas-sensing properties. *J. Mater. Chem. A* **3,** 12796–12803 (2015).

22. Serre, C. et al. Role of solvent-host interactions that lead to very large swelling of hybrid frameworks. *Science* **315,** 1828–1832 (2007).
