# Supplementary material for: Computer-aided discovery of connected metal-organic frameworks
Source: Nat Commun. 2019 Aug 9;10:3620. doi: 10.1038/s41467-019-11629-4 (PMC6689093; doi:10.1038/s41467-019-11629-4)
Supplement: Supplementary file 2 — Description of Additional Supplementary Files [file 41467_2019_11629_MOESM2_ESM.docx]

Description of Additional Supplementary Files

**Supplementary Data 1:** Computational screening result of test case (MOF-5) and general screening results.
